# Association of Preeclampsia and Perinatal Complications With Offspring Neurodevelopmental and Psychiatric Disorders

**DOI:** 10.1001/jamanetworkopen.2021.45719

**Published:** 2022-01-28

**Authors:** Linghua Kong, Xinxia Chen, Yajun Liang, Yvonne Forsell, Mika Gissler, Catharina Lavebratt

**Affiliations:** 1 Department of Molecular Medicine and Surgery, Karolinska Institutet, Stockholm, Sweden; 2 Translational Psychiatry Unit, Centre for Molecular Medicine, Karolinska University Hospital, Stockholm, Sweden; 3 School of Nursing and Rehabilitation, Cheeloo College of Medicine, Shandong University, Shandong, China; 4 Department of Global Public Health, Karolinska Institutet, Stockholm, Sweden; 5 Department of Information Services, Finnish Institute for Health and Welfare, Helsinki, Finland

## Abstract

**Importance:**

Maternal preeclampsia has been reported to increase the risk of autism spectrum disorder, attention-deficit/hyperactivity disorder (ADHD), and intellectual disability in offspring. However, the association between maternal preeclampsia combined with perinatal complications and neurodevelopmental and psychiatric disorders in offspring is less well documented.

**Objective:**

To examine the association of maternal preeclampsia, separately and together with perinatal complications, with neurodevelopmental and psychiatric disorders in offspring.

**Design, Setting, and Participants:**

This population-based cohort study used data from nationwide registries in Finland to assess all singleton live births (N = 1 012 723) between January 1, 1996, and December 31, 2014. Offspring were followed up until December 31, 2018 (when the oldest reached age 22 years). Exclusion criteria were maternal inpatient psychiatric diagnoses and pregestational diabetes. The study and data analysis were conducted from May 1, 2020, to June 1, 2021.

**Exposures:**

Preeclampsia and perinatal complications (delivery earlier than 34 weeks’ gestation and/or small for gestational age).

**Main Outcomes and Measures:**

The primary outcomes were neurodevelopmental and psychiatric diagnoses and dispensation of psychotropic drugs among offspring until December 31, 2018. Cox proportional hazards regression analyses were performed to assess the associations.

**Results:**

Of 1 012 723 singleton live births (51.1% boys; mean [SD] maternal age at birth, 30.0 [5.4] years; specific data on race and ethnicity were not available in the data set), 21 010 children (2.1%) were exposed to preeclampsia alone, 33 625 children (3.3%) were exposed to perinatal complications alone, and 4891 children (0.5%) were exposed to both preeclampsia and perinatal complications. A total of 93 281 children (9.2%) were diagnosed with a neurodevelopmental or psychiatric disorder. Offspring exposed to both preeclampsia and perinatal complications had an increased risk of any neurodevelopmental or psychiatric disorder after adjusting for potential confounding (adjusted hazard ratio [aHR], 2.11; 95% CI, 1.96-2.26) compared with those not exposed to either preeclampsia or perinatal complications; this risk was higher than exposure to either preeclampsia alone (aHR, 1.18; 95% CI, 1.12-1.23) or perinatal complications alone (aHR, 1.77; 95% CI, 1.72-1.82). Sibling pair analyses did not detect any increase in the risk of neurodevelopmental or psychiatric disorders after exposure to preeclampsia alone, but offspring exposed to both preeclampsia and perinatal complications had increased risks of intellectual disabilities (aHR, 3.24; 95% CI, 1.05-10.06), specific developmental disorders (aHR, 3.56; 95% CI, 2.35-5.41), ADHD and conduct disorders (aHR, 2.42; 95% CI, 1.09-5.39), and other behavioral and emotional disorders (aHR, 2.45; 95% CI, 1.17-5.13). The risk estimates for specific developmental disorders (aHR, 2.82; 95% CI, 2.60-3.05) and ADHD and conduct disorders (aHR, 1.88; 95% CI, 1.65-2.14) were higher among offspring exposed to both preeclampsia and perinatal complications compared with those exposed to perinatal complications alone (aHR, 2.26 [95% CI, 2.18-2.33] and 1.60 [95% CI, 1.52-1.68], respectively).

**Conclusions and Relevance:**

In this study, exposure to both maternal preeclampsia and perinatal complications was associated with intellectual disabilities, specific developmental disorders, ADHD and conduct disorders, and other behavioral and emotional disorders in offspring. For specific developmental disorders and ADHD and conduct disorders, the risk estimates were higher among offspring exposed to both preeclampsia and perinatal complications compared with those exposed to perinatal complications only.

## Key Points

**Question:**

Is maternal preeclampsia, alone or together with perinatal complications (preterm birth and/or small birth size) associated with an increased risk of neurodevelopmental and psychiatric disorders in offspring?

**Findings:**

In this cohort study of 1 012 723 singleton live births in Finland, exposure to both maternal preeclampsia and perinatal complications was associated with higher risks of specific neurodevelopmental disorders as well as attention-deficit/hyperactivity disorder and conduct disorders in offspring compared with exposure to either preeclampsia or perinatal complications alone.

**Meaning:**

These results suggest that children exposed to both preeclampsia in utero and perinatal complications have modestly increased risks of developing specific neurodevelopmental disorders as well as attention-deficit/hyperactivity disorder and conduct disorders.

## Teaser

This cohort study uses data from national registers in Finland to examine the association between exposure to maternal preeclampsia, alone and together with exposure to perinatal complications, and the risk of neurodevelopmental and psychiatric disorders in offspring.

## Introduction

Preeclampsia, occurring in 3% to 5% of pregnancies worldwide, is characterized by new-onset hypertension along with proteinuria after 20 weeks’ gestation and is often accompanied by uteroplacental dysfunction with abnormal blood vessel development and other maternal organ dysfunction.^1,2,3^ Preeclampsia is a major factor associated with maternal and perinatal morbidity and mortality associated with eclampsia, stroke, and kidney failure as well as hemolysis, elevated liver enzymes, and low platelet syndrome.^4^ In addition, preeclampsia has been associated with long-term endocrine and cardiovascular morbidity in offspring.^5,6,7^ However, preeclampsia may present with or without severe features. Although diagnostic criteria for severe preeclampsia are included in the *International Classification of Diseases, Tenth Revision* (*ICD-10*), recent clinical care guidelines recommend avoiding early classification of preeclampsia as mild or severe because the condition can deteriorate rapidly.^3^ Given that delivery is the only effective treatment for preeclampsia, delivery before 34 weeks’ gestation is often used as a retrospective proxy for severe preeclampsia.^8^ In addition, because small for gestational age (SGA) status at birth is associated with uteroplacental dysfunction, preeclampsia combined with SGA status is also considered a severe condition.^9,10^

Preeclampsia has been associated with increased risks of several neurodevelopmental disorders in offspring, including autism spectrum disorder (ASD), attention-deficit/ hyperactivity disorder (ADHD), schizophrenia, intellectual disability, epilepsy, and cerebral palsy.^9,10,11,12,13,14,15^ Several systematic reviews^16,17,18^ reported that preeclampsia was associated with increases of 50% in the risk of ASD, 30% in the risk of ADHD, and 40% in the risk of schizophrenia among offspring. In 1 meta-analysis,^18^ only a few studies of ASD and ADHD assessed familial confounding by including siblings. A recent study^19^ in Finland followed up 4743 offspring to age 10 years and grouped offspring diagnoses, finding that maternal preeclampsia, but not other maternal hypertensive disorders, was associated with an increased risk of both psychological developmental disorders (*ICD-10* codes F80-F89, with code F84 indicating ASD) and emotional and behavioral disorders (*ICD-10* codes F90-F98, with code F90 indicating ADHD). This study^19^ also found effect sizes had a propensity to be larger for severe preeclampsia (*ICD-10* code O14.1) than for mild to moderate preeclampsia (*ICD-10* code O14.0) and suggested through findings from hierarchical regression analyses that maternal and paternal mental disorders did not have an impact for these effect sizes. Additive consequences were also detected, with a higher number of maternal metabolic and hypertensive disorders (including obesity, diabetes, and hypertension; 0-3 disorders) being associated with a greater risk of neuropsychiatric diagnoses in children. Furthermore, the authors suggested that preterm birth and SGA status partially mediated some of the associations between preeclampsia and child diagnoses.^19^ These findings were consistent with those of similar studies included in a systematic review.^18^

In the present cohort study, we extended these findings with the aim of assessing the effect sizes of exposure to maternal preeclampsia together with perinatal complications for a wide range of individual neurodevelopmental and psychiatric diagnoses among offspring followed up to age 22 years. We used a recommended indicator of severe preeclampsia that was based on preterm birth (earlier than 34 weeks’ gestation) and/or SGA status,^8,9,10^ and we compared the effect sizes of exposure to both preeclampsia and perinatal complications with those of exposure to preeclampsia or perinatal complications alone. Moreover, we performed sibling analyses to examine whether detected associations could be explained by familial confounding. To conduct these analyses, we used data from more than 1 million live births recorded in nationwide registers in Finland and followed up offspring to age 22 years.

## Methods

### Study Population and Data Sources

This population-based cohort study included all 1 012 723 singleton live births in Finland from January 1, 1996, to December 31, 2014, that were recorded in the Drugs and Pregnancy Database,^20,21^ which contains data from the Medical Birth Register,^22,23^ the Register on Induced Abortions,^24,25^ and the Register of Congenital Malformations,^26^ all of which are maintained by the Finnish Institute for Health and Welfare.^20^ Registers are described in eMethods in the Supplement. This study was approved by the Drugs and Pregnancy Database steering committee and the data protection authority in Finland. Register linkages were conducted as specified in the agreement between the register administrators (the Social Insurance Institution of Finland and the Finnish Institute for Health and Welfare). Data were obtained from Finnish administrative registers. According to Finnish law, informed consent is not required for the use of data from these registers. All data were deidentified, and no registered person (mother or child) was contacted. This study followed the Strengthening the Reporting of Observational Studies in Epidemiology (STROBE) reporting guideline for cohort studies.

The study and data analysis were conducted from May 1, 2020, to June 1, 2021. All offspring were followed up until December 31, 2018 (when the oldest reached age 22 years). The exclusion criteria were maternal pregestational diabetes (n = 25 901) and maternal in-hospital psychiatric history (n = 20 486) before or during pregnancy because these exposures could have increased the risks of neurodevelopmental and psychiatric disorders in offspring, with moderate to high effect sizes.^27,28^ In all analyses, with the exception of sibling analyses, mothers with chronic hypertension (n = 16 434) and gestational hypertension (n = 17 469) were excluded from the group with no exposure to either preeclampsia or perinatal complications (reference group) because they could have produced bias in the main results.^9,10^

### Main Exposures

Main exposures included maternal preeclampsia (identified through *ICD-10* code O11 [14 726 participants] or O14 [11 175 participants] in the Finnish Care Registers for Health Care^29^) and perinatal complications, including SGA status and/or delivery earlier than 34 weeks’ gestation (identified through the Drugs and Pregnancy Database). Small for gestational age was defined as birth weight and/or length more than 2 SDs lower than the sex-specific and gestational age–specific mean in the Finnish population^30^ based on criteria from the International Societies of Pediatric Endocrinology and the Growth Hormone Research Society.^31^

### Outcomes and Covariates

Offspring neurodevelopmental and psychiatric disorders between 1996 and 2018, as defined by *ICD-10* codes from the Finnish Care Registers for Health Care, were used as outcome variables. These variables included psychotic disorders (*ICD-10* codes F20-F29), mood disorders (*ICD-10* codes F30- F39 and F92), anxiety disorders (*ICD-10* codes F40-F43 and F93), eating disorders (*ICD-10* code F50), sleeping disorders (*ICD-10* code F51), personality disorders (*ICD-10* codes F60-F69), intellectual disabilities (*ICD-10* codes F70-F79), specific developmental disorders (*ICD-10* codes F80-F83), ASD (*ICD-10* code F84), ADHD and conduct disorders (*ICD-10* codes F90 and F91), and other behavioral and emotional disorders (*ICD-10* code F98) (eTable 1 in the Supplement). Data on dispensation of psychotropic drugs prescribed to offspring were extracted using Anatomical Therapeutic Chemical (ATC) classification system codes from the Finnish Register on Reimbursement Drugs. Drugs included antipsychotic, anxiolytic, hypnotic, and sedative medications (ATC group N05); antidepressant medications (ATC group N06A); and psychostimulant and nootropic medications (ATC group N06B).

The covariates included offspring birth year, offspring sex, and maternal factors, including age at delivery, country of birth (Finland or other), married at birth (yes or no), occupation (upper white collar worker, lower white collar worker, blue collar worker, or other status), smoking status (yes or no), parity (0 or ≥1 births to a fetus with gestational age ≥24 weeks, regardless of whether the child was born alive or stillborn) identified through the Drugs and Pregnancy Database, obesity (*ICD-10* codes E65 and E66; yes or no), gestational diabetes (yes or no), outpatient psychiatric disorders (*ICD-10* codes F00-F99; yes or no), systemic inflammatory disease (*ICD-10* codes M30-M36; yes or no) identified through the Finnish Care Registers for Health Care, use of psychotropic medication during pregnancy (yes or no) identified through the Finnish Register on Reimbursement Drugs, and interval between pregnancies.

### Statistical Analysis

Cox proportional hazards modeling was used to examine the association of maternal preeclampsia and perinatal complications with the diagnosis of neurodevelopmental and psychiatric disorders in offspring and the dispensation of psychotropic drugs (sensitivity analysis) to offspring after adjusting for potential confounding. The proportional hazards assumption was tested.

Sibling pair analyses were also performed to investigate whether any associations between exposure to maternal preeclampsia with perinatal complications and neurodevelopmental and psychiatric disorders in offspring were explained by familial confounding. All singleton sibling pairs from consecutive pregnancies were included. In a sensitivity analysis, only the first 2 subsequent singleton pregnancies of the same mother during the study period were included. The risk of neuropsychiatric disorders for the second (younger) sibling was estimated after stratifying for both the first and second siblings’ exposure or nonexposure to preeclampsia and/or perinatal complications. The reference group comprised unexposed second siblings with first siblings who were also unexposed. Second siblings were followed up for psychiatric diagnosis outcomes and dispensation of psychotropic medication until December 31, 2018. Exposure-based sibling pair stratification allowed us to calculate the risk estimates for outcomes among exposed second siblings and compare those results with risk estimates among unexposed second siblings who had exposed older siblings. In model 2, the first sibling was also followed up for outcomes. Model 2 was further adjusted for the corresponding psychiatric diagnosis or dispensation of psychotropic medication for the first sibling, irrespective of the exposure to the first sibling, as an attempt to reduce genetic confounding. Overall, this sibling pair analysis approach enabled detection of exposure-specific associations among second siblings that were not explained by exposure in the first siblings only, thereby excluding complete familial confounding.

Hazard ratios (HRs) with 95% CIs were reported for the risks of neurodevelopmental and psychiatric outcomes. Two-sided *P* < .05 was considered statistically significant. All statistical analyses were performed using SAS software, version 9.4 (SAS Institute Inc).

## Results

**Figure 1.**
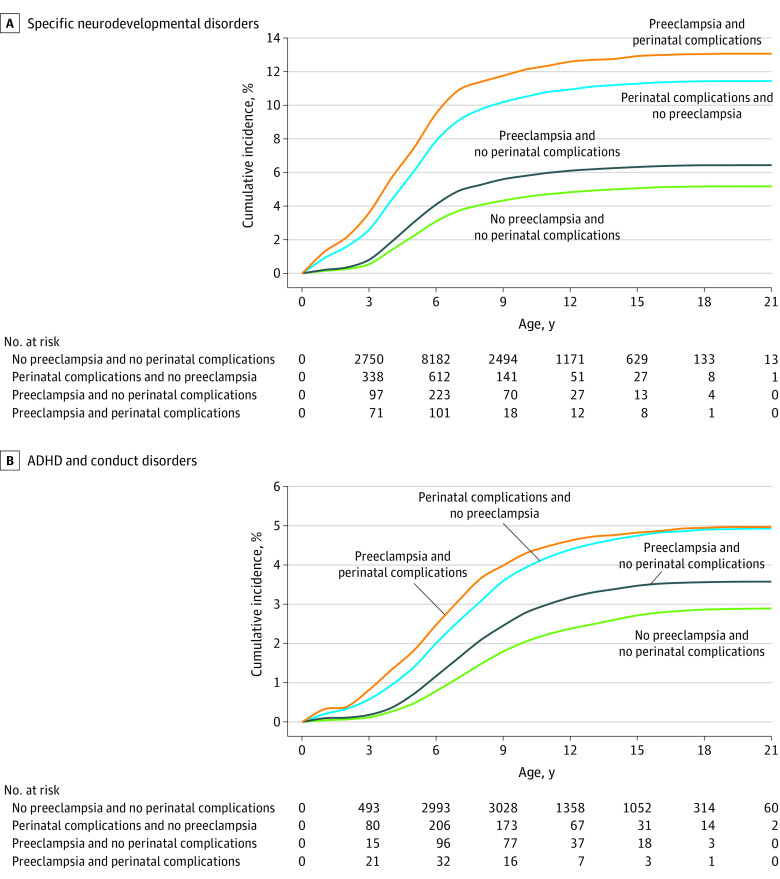
Maternal Preeclampsia and Incidence of Neurodevelopmental and Psychiatric Disorders in Offspring Preeclampsia was defined as *International Classification of Diseases, Tenth Revision* (*ICD-10*), diagnostic code O11 or O14. Perinatal complications were defined as small for gestational age (defined as birth weight and/or length more than 2 SDs lower than the sex-specific and gestational age–specific mean of the Finnish population^1^ based on criteria from the International Societies of Pediatric Endocrinology and the Growth Hormone Research Society^2^) and/or birth earlier than 34 weeks’ gestation. All offspring were followed up until December 2018. A, Specific developmental disorders include *ICD-10* codes F80 to F83. B, Attention-deficit/hyperactivity disorder (ADHD) and conduct disorders include *ICD-10* codes F90 and F91.

Among 1 012 723 singleton live births, 517 923 (51.1%) were boys, and 494 800 (48.9%) were girls; the mean (SD) maternal age at birth was 30.0 (5.4) years. Specific data on race and ethnicity were not available in the data set. A total of 21 010 children (2.1%) were exposed to preeclampsia alone, 33 625 children (3.3%) were exposed to perinatal complications alone, and 4891 children (0.5%) were exposed to both preeclampsia and perinatal complications (Table 1). Offspring were followed up for a mean (SD) of 12.4 (5.7) years, corresponding to 12.6 million person-years. Overall, 93 281 children (9.2%) were diagnosed with a neurodevelopmental or psychiatric disorder between 1996 and 2018. Specific developmental disorders were most common (55 326 children [5.5%]) followed by anxiety disorders (50 731 children [5.0%]), mood disorders (38 293 children [3.8%]), and ADHD and conduct disorders (30 115 children [3.0%]) (eTable 2 in the Supplement). The cumulative incidences of several neurodevelopmental and psychiatric disorders among offspring exposed to both maternal preeclampsia and perinatal complications were higher than those of offspring exposed to preeclampsia alone and those of offspring not exposed to either preeclampsia or perinatal complications, which was exemplified by the incidence of specific developmental disorders (preeclampsia and perinatal complications: 638 of 4891 children [13.0%]; preeclampsia alone: 1357 of 21 030 children [6.5%]; neither preeclampsia nor perinatal complications: 49 442 of 953 197 children [5.2%]) and ADHD and conduct disorders (preeclampsia and perinatal complications: 239 of 4891 children [4.9%]; preeclampsia alone: 744 of 21 030 children [3.5%]; neither preeclampsia nor perinatal complications: 27 461 of 953 197 children [2.9%]) (Figure 1; eTable 2 and eTable 3 in the Supplement).

**Table 1.** Demographic Characteristics of Singleton Live Births in Finland Between 1996 and 2014 Stratified by Preeclampsia and Perinatal Complications

Variables^a^	No preeclampsia with no perinatal complications, No. (%)^b^^,^^c^	No preeclampsia with perinatal complications, No. (%)	Preeclampsia with no perinatal complications, No. (%)	Preeclampsia with perinatal complications, No. (%)
Total births, No.	953 197	33 625	21 010	4891
Decade of birth				
1996-1999	203 996 (21.4)	7104 (21.1)	2956 (14.1)	855 (17.5)
2000-2009	493 804 (51.8)	17 466 (51.9)	11 956 (56.9)	2650 (54.2)
2010-2014	255 397 (26.8)	9055 (26.9)	6098 (29.0)	1386 (28.3)
Offspring sex				
Boy	487 209 (51.1)	17 632 (52.4)	10 680 (50.8)	2402 (49.1)
Girl	465 988 (48.9)	15 993 (47.6)	10 330 (49.2)	2489 (50.9)
Maternal age, y				
<20	23 530 (2.5)	1371 (4.1)	682 (3.2)	133 (2.7)
20-24	152 166 (16.0)	6249 (18.6)	3957 (18.8)	759 (15.5)
25-29	306 375 (32.1)	10 031 (29.8)	6519 (31.0)	1365 (27.9)
30-34	299 677 (31.4)	9661 (28.7)	5897 (28.1)	1387 (28.4)
≥35	171 448 (18.0)	6313 (18.8)	3955 (18.8)	1247 (25.5)
Parity^d^				
0	379 725 (39.8)	19 094 (56.8)	12 721 (60.5)	3257 (66.6)
1	328 751 (34.5)	8179 (24.3)	5303 (25.2)	927 (19.0)
2	148 996 (15.6)	3747 (11.1)	1816 (8.6)	413 (8.4)
3	50 877 (5.3)	1441 (4.3)	618 (2.9)	148 (3.0)
4 or more	43 833 (4.6)	1137 (3.4)	512 (2.4)	142 (2.9)
Missing	1015 (0.1)	27 (0.1)	40 (0.2)	4 (0.1)
Maternal occupation				
Upper white collar	161 147 (16.9)	4810 (14.3)	3347 (15.9)	778 (15.9)
Lower white collar	340 383 (35.7)	11 248 (33.5)	7750 (36.9)	1834 (37.5)
Blue collar	136 880 (14.4)	5490 (16.3)	2955 (14.1)	715 (14.6)
Other	165 209 (17.3)	6132 (18.2)	3404 (16.2)	720 (14.7)
Missing	149 578 (15.7)	5945 (17.7)	3554 (16.9)	844 (17.3)
Mother’s marital status				
Married	571 707 (60.0)	17 424 (51.8)	11 599 (55.2)	2548 (52.1)
Cohabiting	275 008 (28.9)	10 829 (32.2)	6934 (33.0)	1642 (33.6)
Other	88 238 (9.3)	4503 (13.4)	2179 (10.4)	601 (12.3)
Missing	18 244 (1.9)	869 (2.6)	298 (1.4)	100 (2.0)
Mother’s country of birth				
Finland	875 408 (91.8)	30 019 (89.3)	19 824 (94.4)	4516 (92.3)
Other	77 789 (8.2)	3606 (10.7)	1186 (5.6)	375 (7.7)
Maternal smoking				
No	793 268 (83.2)	23 896 (71.1)	17 896 (85.2)	4027 (82.3)
Stopped in first trimester	34 953 (3.7)	1250 (3.7)	967 (4.6)	168 (3.4)
Continued during pregnancy	101 010 (10.6)	7455 (22.2)	1674 (8.0)	525 (10.7)
Missing	23 966 (2.5)	1024 (3.0)	473 (2.3)	171 (3.5)
Maternal systemic inflammatory disease^e^				
Yes	9566 (1.0)	478 (1.4)	271 (1.3)	83 (1.7)
No	943 631 (99.0)	33 147 (98.6)	20 739 (98.7)	4808 (98.3)
Maternal psychiatric outpatient history (1998-2014)				
Yes	59 647 (6.3)	2971 (8.8)	1888 (9.0)	436 (8.9)
No	893 550 (93.7)	30 654 (91.2)	19 122 (91.0)	4455 (91.1)
Maternal receipt of psychotropic medication during pregnancy^f^				
Yes	36 997 (3.9)	1627 (4.8)	1070 (5.1)	222 (4.5)
No	916 200 (96.1)	31 998 (95.2)	19 940 (94.9)	4669 (95.5)
Maternal chronic hypertension^g^				
Yes	0	0	1973 (9.4)	676 (13.8)
No	953 197 (100.0)	33 625 (100.0)	19 037 (90.6)	4215 (86.2)
Maternal gestational hypertension^h^				
Yes	0	0	2966 (14.1)	615 (12.6)
No	953 197 (100.0)	33 625 (100.0)	18 044 (85.9)	4276 (87.4)
Maternal obesity^i^				
Yes	21 801 (2.3)	630 (1.9)	1072 (5.1)	212 (4.3)
No	931 396 (97.7)	32 995 (98.1)	19 938 (94.9)	4679 (95.7)
Maternal gestational diabetes^j^				
Yes	129 921 (13.6)	3214 (9.6)	4863 (23.1)	692 (14.1)
No	823 276 (86.4)	30 411 (90.4)	16 147 (76.9)	4199 (85.9)

^a^ Mothers with in-hospital psychiatric disorders and pregestational diabetes were excluded.

^b^ Preeclampsia based on *International Classification of Diseases, Tenth Revision* (*ICD-10*), diagnostic code O11 or O14.

^c^ Perinatal complications defined as small for gestational age (defined as birth weight and/or length more than 2 SDs lower than the sex-specific and gestational age–specific mean of the Finnish population^1^ based on criteria from the International Societies of Pediatric Endocrinology and the Growth Hormone Research Society^2^) and/or delivery earlier than 34 weeks’ gestation.

^d^ Parity defined as number of births to a fetus with gestational age of 24 weeks or more, regardless of whether the child was born alive or stillborn.

^e^ Maternal systemic inflammatory disease based on *ICD-10* codes M30 to M36.

^f^ Maternal use of psychotropic medications during pregnancy based on Anatomical Therapeutic Chemical classification system codes N05 and N06.

^g^ Maternal chronic hypertension based on *ICD-10* codes I10 to I13 and O10.

^h^ Maternal gestational hypertension based on *ICD-10* code O13.

^i^ Maternal obesity based on *ICD-10* codes E65 and E66.

^j^ Maternal gestational diabetes based on *ICD-10* code O24.4.

**Figure 2.**
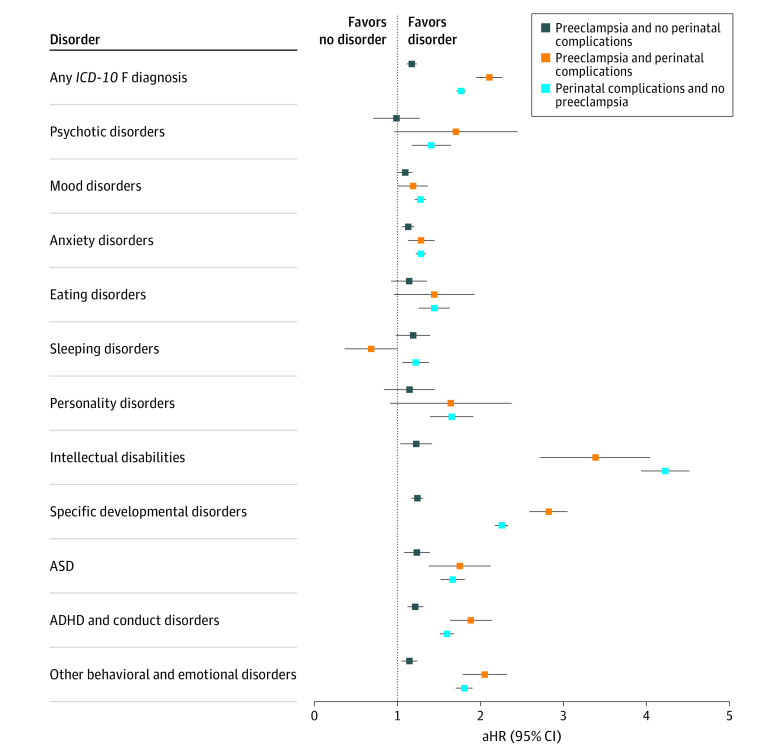
Association of Maternal Preeclampsia and Perinatal Complications With Risks of Neurodevelopmental and Psychiatric Disorders in Offspring Reference group comprised offspring who were not exposed to preeclampsia (*International Classification of Diseases, Tenth Revision* [*ICD-10*], diagnostic code O11 or O14) or perinatal complications (defined as small for gestational age [defined as birth weight and/or length more than 2 SDs lower than the sex-specific and gestational age–specific mean of the Finnish population^1^ based on criteria from the International Societies of Pediatric Endocrinology and the Growth Hormone Research Society^2^] and/or birth at <34 weeks’ gestation) after excluding maternal chronic hypertension and gestational hypertension. Analyses were adjusted for offspring birth year, offspring sex, and maternal factors, including age at delivery, country of birth (Finland or other), married at birth (yes or no), occupation (upper white collar worker, lower white collar worker, blue collar worker, or other status), smoking (yes or no), parity (0 or ≥1 births to a fetus with gestational age ≥24 weeks, regardless of whether the child was born alive or stillborn), obesity (*ICD-10* codes E65 and E66; yes or no), gestational diabetes (yes or no), outpatient psychiatric disorders (yes or no), dispensation of psychotropic medication (Anatomical Therapeutic Chemical classification system codes N05 and N06; yes or no), and systemic inflammatory disease (yes or no). All offspring were followed up until December 2018. ADHD indicates attention-deficit/hyperactivity disorders; aHR, adjusted hazard ratio; ASD, autism spectrum disorders.

Compared with offspring unexposed to preeclampsia and perinatal complications, those exposed to preeclampsia alone had an 18% higher likelihood (adjusted HR [aHR], 1.18; 95% CI, 1.12-1.23) of any neuropsychiatric disorder after adjusting for potential confounding, whereas the aHR for neuropsychiatric disorders among offspring exposed to perinatal complications alone was 1.77 (95% CI, 1.72-1.82) (Figure 2; eTable 4 in the Supplement). Exposure to preeclampsia alone was associated with an increased risk of all neuropsychiatric disorders (ranging from an aHR of 1.10 [95% CI, 1.02-1.18] for mood disorders to 1.24 [95% CI, 1.18-1.31] for specific developmental disorders), with the exception of psychotic disorders (aHR, 0.97; 95% CI, 0.73-1.28), eating disorders (aHR, 1.13; 95% CI, 0.94-1.36), and personality disorders (aHR, 1.12; 95% CI, 0.86-1.46). Exposure to perinatal complications alone was also associated with an increased risk of all neurodevelopmental and psychiatric disorders, with aHRs ranging from 1.22 (95% CI, 1.07-1.38) for sleeping disorders to 4.22 (95% CI, 3.95-4.52) for intellectual disabilities (Figure 2; eTable 4 in the Supplement). A similar pattern was observed for unadjusted HRs (eTable 4 in the Supplement).

Offspring exposed to both preeclampsia and perinatal complications had a more than 2-fold risk of developing any neurodevelopmental or psychiatric disorder (aHR, 2.11; 95% CI, 1.96-2.26), which was higher than the risk of those exposed to either preeclampsia alone (aHR, 1.18; 95% CI, 1.12-1.23) or perinatal complications alone (aHR, 1.77; 95% CI, 1.72-1.82). Exposure to both preeclampsia and perinatal complications was also associated with specific developmental disorders (aHR, 2.82; 95% CI, 2.60-3.05) and ADHD and conduct disorders (aHR, 1.88; 95% CI, 1.65-2.14); these risk estimates were higher than exposure to preeclampsia alone (specific developmental disorders: aHR, 1.24 [95% CI, 1.18-1.31]; ADHD and conduct disorders: aHR, 1.22 [95% CI, 1.13-1.31]) and perinatal complications alone (specific developmental disorders: aHR, 2.26 [95% CI, 2.18-2.33]; ADHD and conduct disorders: aHR, 1.60 [95% CI, 1.52-1.68]) (Figure 2; eTable 4 in the Supplement). In addition, exposure to both preeclampsia and perinatal complications vs exposure to preeclampsia alone was associated with a higher risk of psychotic disorders (aHR, 1.60 [95% CI, 1.03-2.49] vs 0.97 [95% CI, 0.73-1.28]), anxiety disorders (aHR, 1.28 [95% CI, 1.14-1.45] vs 1.13 [95% CI, 1.06-1.20]), intellectual disabilities (aHR, 3.34 [95% CI, 2.75-4.06] vs 1.22 [95% CI, 1.05-1.42]), ASD (aHR, 1.73 [95% CI, 1.40-2.13] vs 1.23 [95% CI, 1.09-1.39]) and other behavioral and emotional disorders (aHR, 2.04 [95% CI, 1.80-2.32] vs 1.14 [95% CI, 1.06-1.24]). However, the risk estimates for exposure to both preeclampsia and perinatal complications were equal to or lower than exposure to perinatal complications only (psychotic disorders: aHR, 1.40 [95% CI, 1.19-1.65]; anxiety disorders: aHR, 1.28 [95% CI, 1.23-1.34]; intellectual disabilities: aHR, 4.22 [95% CI, 3.95-4.52]; ASD: aHR, 1.67 [95% CI, 1.53-1.81]; other behavioral and emotional disorders: aHR, 1.81 [95% CI, 1.71-1.90]) (Figure 2; eTable 4 in the Supplement). No association was found between exposure to both preeclampsia and perinatal complications and eating disorders (aHR, 1.40; 95% CI, 0.99-1.95), sleeping disorders (aHR, 0.64; 95% CI, 0.40-1.02), or personality disorders (aHR, 1.54; 95% CI, 0.98-2.42), whereas exposure to perinatal complications alone was associated with a higher risk of eating disorders (aHR, 1.44; 95% CI, 1.27-1.63), sleeping disorders (aHR, 1.22; 95% CI, 1.07-1.38), and personality disorders (aHR, 1.64; 95% CI, 1.41-1.92) (Figure 2; eTable 4 in the Supplement).

A total of 50 131 singleton live offspring (5.0%) received prescribed psychotropic medications, including antipsychotic and hypnotic or anxiolytic drugs (ATC group N05; 33 471 children), antidepressant drugs (ATC group N06A; 11 509 children), and stimulant drugs (ATC group N06B; 14 547 children) (eTable 5 in the Supplement**)**. We examined the association between the exposures and the dispensation of psychotropic medications as an estimate of offspring neuropsychiatric disorders using a sensitivity analysis. After adjusting for potential confounding, exposure to preeclampsia only was associated with slightly higher risks of dispensation of any psychotropic medication (aHR, 1.09; 95% CI, 1.02-1.17) and stimulant medication (aHR, 1.25; 95% CI, 1.12-1.40). Exposure to both preeclampsia and perinatal complications was associated with a higher risk of dispensation of any psychotropic medication (aHR, 1.52; 95% CI, 1.35-1.71); antipsychotic, anxiolytic, hypnotic, and sedative drugs (aHR, 1.53; 95% CI, 1.33-1.76); antidepressant drugs (aHR, 1.34; 95% CI, 1.02-1.75); and stimulant drugs (aHR, 1.64; 95% CI, 1.33-2.02); however, the effect sizes were similar to those among offspring exposed to perinatal complications only (any psychotropic drug: aHR, 1.53 [95% CI, 1.47-1.59]; antipsychotic, anxiolytic, hypnotic, and sedative drugs: aHR, 1.55 [95% CI, 1.47-1.63]; antidepressant drugs: aHR, 1.25 [95% CI, 1.14-1.37]; stimulant drugs: aHR, 1.69 [95% CI, 1.57-1.81]) (eFigure 1 in the Supplement).

The results of sibling pair analyses suggested that the associations detected between exposure to both preeclampsia and perinatal complications and specific developmental disorders, ADHD and conduct disorders, intellectual disabilities, and other behavioral and emotional disorders were not confounded (Table 2; eTable 6 in the Supplement), and the associations with other neuropsychiatric diagnoses were explained by within-pair shared familial factors (eTable 7 and eTable 8 in the Supplement). The effect size for the risk of any neuropsychiatric diagnosis (ie, any *ICD-10* F code) among second siblings who had unexposed first siblings (aHR, 2.02; 95% CI, 1.66-2.45) was similar to the effect size for risk in the whole cohort (aHR, 2.11; 95% CI, 1.96-2.26); however, when only the first sibling was exposed, the second sibling had no increased risk of any neuropsychiatric diagnosis (aHR, 0.90; 95% CI, 0.74-1.09) (Table 2). When both siblings in the pair were exposed to both preeclampsia and perinatal complications, the risk of any neuropsychiatric diagnosis was larger (aHR, 3.19; 95% CI, 2.14-4.77).

**Table 2.** Sibling Pair Analysis of Exposure to Both Maternal Preeclampsia and Perinatal Complications^a^

Exposure to preeclampsia with perinatal complications^c^	Total sibling pairs, No.	aHR (95% CI)^b^				
		Any neuropsychiatric diagnosis^d^	Intellectual disabilities^e^	Specific developmental disorders^f^	ADHD and conduct disorders^g^	Other behavioral and emotional disorders^h^
Model 1^i^						
Neither child/pregnancy in sibling pair exposed	435 997	1.0 [Reference]	1.0 [Reference]	1.0 [Reference]	1.0 [Reference]	1.0 [Reference]
First but not second child/pregnancy exposed	1614	0.96 (0.79-1.17)	0.72 (0.36-1.43)	1.02 (0.82-1.27)	0.93 (0.66-1.29)	1.04 (0.76-1.42)
Second but not first child/pregnancy exposed	873	2.08 (1.71-2.53)	3.17 (2.02-4.98)	2.82 (2.34-3.39)	1.81 (1.26-2.59)	1.52 (1.05-2.22)
Both children/pregnancies in sibling pair exposed	142	3.34 (2.24-5.00)	3.40 (1.10-10.55)	3.72 (2.45-5.64)	2.51 (1.13-5.59)	2.53 (1.21-5.30)
Model 2^j^						
Neither child/pregnancy in sibling pair exposed	435 997	1.0 [Reference]	1.0 [Reference]	1.0 [Reference]	1.0 [Reference]	1.0 [Reference]
First but not second child/pregnancy exposed	1614	0.90 (0.74-1.09)	0.66 (0.33-1.33)	0.95 (0.76-1.18)	0.86 (0.62-1.19)	0.98 (0.72-1.34)
Second but not first child/pregnancy exposed	873	2.02 (1.66-2.45)	3.05 (1.95-4.79)	2.71 (2.25-3.26)	1.73 (1.21-2.48)	1.49 (1.02-2.18)
Both children/pregnancies in sibling pair exposed	142	3.19 (2.14-4.77)	3.24 (1.05-10.06)	3.56 (2.35-5.41)	2.42 (1.09-5.39)	2.45 (1.17-5.13)

Abbreviations: ADHD, attention-deficit/hyperactivity disorders; aHR, adjusted hazard ratio.

^a^ Sibling pair analysis of all 438 626 consecutive sibling pairs among 1 012 723 births in Finland between 1996 and 2014. All mothers with a singleton sibling pair were included. Mothers with in-hospital psychiatric disorders and pregestational diabetes were excluded.

^b^ The aHRs for outcomes in the second child included diagnosis of any neurodevelopmental or psychiatric disorder (all F diagnostic codes from the *International Classification of Diseases, Tenth Revision* [*ICD-10*]) with regard to exposure of the sibling pair to preeclampsia with perinatal complications.

^c^ Reference group comprised offspring who were not exposed to maternal preeclampsia (defined as *International Classification of Diseases, Tenth Revision* [*ICD-10*] code O11 or O14) or perinatal complications (defined as birth weight and/or length more than 2 SDs lower than the sex-specific and gestational age–specific mean of the Finnish population^1^ based on criteria from the International Societies of Pediatric Endocrinology and the Growth Hormone Research Society^2^ and/or delivery earlier than 34 weeks’ gestation).

^d^ Includes all *ICD-10* F codes.

^e^ Includes *ICD-10* codes F70 to F79.

^f^ Includes *ICD-10* codes F80 to F83.

^g^ Includes *ICD-10* codes F90 and F91.

^h^ Includes *ICD-10* code F98.

^i^ Model 1 was adjusted for offspring birth year, offspring sex, and maternal factors, including age at delivery, country of birth (Finland or other country), married at birth (yes or no), occupation (upper white collar worker, lower white collar worker, blue collar worker, or other status), smoking (yes or no), parity (0 or ≥1 births to a fetus with gestational age ≥24 weeks, regardless of whether the child was born alive or stillborn), obesity (*ICD-10* codes E65 and E66; yes or no), gestational diabetes (yes or no), outpatient psychiatric disorders (yes or no), dispensation of psychotropic medication (Anatomical Therapeutic Chemical classification system codes N05 and N06; yes or no) during pregnancy (yes or no), systemic inflammatory disease (yes or no), and intrapregnancy interval.

^j^ Model 2 was adjusted for all variables in model 1 plus the presence of a corresponding neurodevelopmental or psychiatric disorder or the dispensation of psychotropic medication to the first child.

Sibling pair analysis of the effect sizes of exposure to perinatal complications alone revealed that the detected association between perinatal complications and the risk of any neuropsychiatric diagnosis was not explained only by familial confounding. When both siblings in the pair were exposed, the effect sizes for the risks of specific developmental disorders (aHR, 2.40; 95% CI, 2.09-2.76) and ADHD and conduct disorders (aHR, 2.14; 95% CI, 1.69-2.71) (Table 3) among those exposed to perinatal complications alone were smaller than the effect sizes among those exposed to both preeclampsia and perinatal complications (specific developmental disorders: aHR, 3.56 [95% CI, 2.35-5.41]; ADHD and conduct disorders: aHR, 2.42 [95% CI, 1.09-5.39]) (Table 2), which were consistent with the risk estimates for the whole cohort (specific developmental disorders: aHR, 2.26 [95% CI, 2.18-2.33]; ADHD and conduct disorders: aHR, 1.60 [95% CI, 1.52-1.68]) (Figure 2; eTable 4 in the Supplement). However, the detected associations between exposure to preeclampsia alone and the risk of any neuropsychiatric diagnosis were all explained by familial confounding (eTable 9 in the Supplement).

**Table 3.** Sibling Pair Analysis of Exposure to Perinatal Complications Only^a^

Exposure to perinatal complications only^c^	Total sibling pairs, No.	aHR (95% CI)^b^		
		Any neuropsychiatric diagnosis^d^	Specific developmental disorders^e^	ADHD and conduct disorders^f^
Model 1^g^				
Neither child/pregnancy in sibling pair exposed	417 922	1.0 [Reference]	1.0 [Reference]	1.0 [Reference]
First but not second child/pregnancy exposed	15 730	1.06 (1.00-1.13)	1.07 (0.99-1.17)	1.04 (0.90-1.19)
Second but not first child/pregnancy exposed	9392	1.96 (1.86-2.08)	2.66 (2.47-2.85)	1.90 (1.65-2.18)
Both children/pregnancies in sibling pair exposed	2483	2.02 (1.82-2.25)	2.61 (2.27-2.99)	2.37 (1.87-3.01)
Model 2^h^				
Neither child/pregnancy in sibling pair exposed	417 922	1.0 [Reference]	1.0 [Reference]	1,0 [Reference]
First but not second child/pregnancy exposed	15 730	1.00 (0.94-1.06)	1.00 (0.92-1.09)	0.95 (0.83-1.10)
Second but not first child/pregnancy exposed	9392	1.91 (1.80-2.02)	2.57 (2.39-2.76)	1.82 (1.58-2.09)
Both children/pregnancies in sibling pair exposed	2483	1.88 (1.68-2.09)	2.40 (2.09-2.76)	2.14 (1.69-2.71)

Abbreviations: ADHD, attention-deficit/hyperactivity disorders; aHR, adjusted hazard ratio.

^a^ Sibling pair analysis of all 445 527 consecutive sibling pairs among 1 012 723 births in Finland between 1996 and 2014. All mothers with a singleton sibling pair were included. Mothers with in-hospital psychiatric disorders, pregestational diabetes, and preeclampsia (*ICD-10* code O11 or O14) were excluded. Sibling pairs with missing information on gestational age or birth weight for either sibling were also excluded.

^b^ The aHRs for outcomes in the second child included diagnosis of any neurodevelopmental or psychiatric disorder (all F diagnostic codes from the *International Classification of Diseases, Tenth Revision* [*ICD-10*]) with regard to exposure of the sibling pair to perinatal complications.

^c^ Reference group comprised offspring who were not exposed to maternal preeclampsia (defined as *International Classification of Diseases, Tenth Revision* [*ICD-10*] code O11 or O14) or perinatal complications (defined as birth weight and/or length more than 2 SDs lower than the sex-specific and gestational age–specific mean of the Finnish population^1^ based on criteria from the International Societies of Pediatric Endocrinology and the Growth Hormone Research Society^2^ and/or delivery earlier than 34 weeks’ gestation).

^d^ Includes all *ICD-10* F codes.

^e^ Includes *ICD-10* codes F80 to F83.

^f^ Includes *ICD-10* codes F90 and F91.

^g^ Model 1 was adjusted for offspring birth year, offspring sex, and maternal factors, including age at delivery, country of birth (Finland or other country), married at birth (yes or no), occupation (upper white collar worker, lower white collar worker, blue collar worker, or other status), smoking (yes or no), parity (0 or ≥1 births to a fetus with gestational age ≥24 weeks, regardless of whether the child was born alive or stillborn), obesity (*ICD-10* codes E65 and E66; yes or no), gestational diabetes (yes or no), outpatient psychiatric disorders (yes or no), dispensation of psychotropic medication (Anatomical Therapeutic Chemical classification system codes N05 and N06; yes or no) during pregnancy (yes or no), systemic inflammatory disease (yes or no), and intrapregnancy interval.

^h^ Model 2 was adjusted for all variables in model 1 plus the presence of a corresponding neurodevelopmental or psychiatric disorder or the dispensation of psychotropic medication to the first child.

We also conducted sensitivity analyses of exposure diagnoses. Rather than using both *ICD-10* codes O14 (preeclampsia) and O11 (preexisting hypertension with preeclampsia) to define preeclampsia, we used only *ICD-10* code O14. The risk estimate pattern was similar to that of *ICD-10* codes O14 plus O11 (eg, risk of any neuropsychiatric diagnosis among those exposed to preeclampsia with perinatal complications: aHR, 2.51 [95% CI, 2.22-2.84] using *ICD-10* code O14 alone vs 2.11 [95% CI, 1.96-2.26] using *ICD-10* codes O14 and O11); however, because the number of mothers in the *ICD-10* code O14 group was smaller (43% of those in the *ICD-10* codes O14 plus O11 group), the 95% CIs were broader (eTable 10 and eTable 11 in the Supplement). Inclusion of mothers with inpatient psychiatric diagnoses and pregestational diabetes, and subsequent adjustment, did not substantially change the effect sizes (eg, risk of any neuropsychiatric diagnosis among those exposed to preeclampsia with perinatal complications: aHR, 2.08; 95% CI, 1.94-2.22]) (eTable 12 in the Supplement). We also estimated effect sizes for the association of exposure to gestational hypertension (*ICD-10* code O13), rather than preeclampsia, with neurodevelopmental and psychiatric disorders. Exposure to both gestational hypertension and perinatal complications vs preeclampsia and perinatal complications was associated with lower effect sizes for specific developmental disorders (aHR, 1.99 [95% CI, 1.65-2.40] vs 2.82 [95% CI, 2.60-3.05]), ASD (aHR, 1.14 [95% CI, 0.66-1.96] vs 1.73 [95% CI, 1.40-2.13]), and other behavioral and emotional disorders (aHR, 1.58 [95% CI, 1.17-2.14] vs 2.04 [95% CI, 1.80-2.32]) and a higher effect size for sleeping disorders (aHR, 3.09 [95% CI, 1.92-4.98] vs 0.64 [95% CI, 0.40-1.02]). All other effect sizes were similar to those found for exposure to both preeclampsia and perinatal complications (eTable 13 in the Supplement).

The mediation analysis revealed that preterm birth and/or SGA status significantly mediated the association between maternal preeclampsia and any neuropsychiatric diagnosis in offspring (total association: HR, 1.05 [95% CI, 1.04-1.07]; direct association: HR, 1.01 [95% CI, 1.00-1.03]; by perinatal complications: HR, 1.04 [95% CI, 1.02-1.07]) (eTable 14 and eFigure 2 in the Supplement).

## Discussion

This cohort study investigated the associations of prenatal exposure to preeclampsia and/or perinatal complications with neurodevelopmental and psychiatric disorders in offspring. Novel approaches of this study included (1) comparison of exposure to both preeclampsia and perinatal complications with exposure to preeclampsia alone and perinatal complications alone and (2) estimation of the risk of a wide spectrum of neurodevelopmental and psychiatric disorders in offspring. Using a large population-based cohort in Finland comprising more than 1 million singleton live births, we found an increased risk of specific developmental disorders (*ICD-10* codes F80-F83), ADHD and conduct disorders (*ICD-10* codes F90 and F91), intellectual disabilities (*ICD-10* codes F70-F79), and other behavior and emotional disorders (*ICD-10* code F98) in offspring exposed to both maternal preeclampsia and perinatal complications. These associations were not explained by measured confounders or unmeasured familial confounders. The risks of specific developmental disorders and ADHD and conduct disorders among offspring exposed to both preeclampsia and perinatal complications were higher than those of offspring exposed to perinatal complications only. The effect sizes for exposure to both preeclampsia and perinatal complications had aHRs ranging from 2 to 3. However, the associations between exposure to preeclampsia alone and offspring neurodevelopmental and psychiatric disorders were explained by unmeasured familial confounding, as revealed in our sibling pair analyses.

Gestational hypertension represents a more benign increase in blood pressure than preeclampsia.^32^ In the present cohort, exposure to both gestational hypertension and perinatal complications was associated with lower effect sizes for the risk of specific developmental disorders, ASD, and other behavioral and emotional disorders compared with exposure to both preeclampsia and perinatal complications. However, exposure to both gestational hypertension and perinatal complications had an unexpectedly higher effect size for the risk of sleeping disorders; however, all other effect sizes were similar to those among offspring exposed to both preeclampsia and perinatal complications, suggesting that this finding warrants further studies.

Meta-analyses^16,17^ have reported that preeclampsia is associated with modest increases in the risk of ASD, ADHD, and schizophrenia. A large population-based cohort study in Sweden that examined the associations between preeclampsia and ASD using a severity indicator found that, among 2 842 230 singleton live births from 1982 to 2010, children exposed to preeclampsia had an increased risk of ASD (aHR, 1.25; 95% CI, 1.19-1.30), and children exposed to preeclampsia who were born SGA had an even higher risk of ASD (aHR, 1.66; 95% CI, 1.49-1.85); however, the risk estimate was comparable with that of children born SGA only.^10^ Another Swedish national register–based study including 2 047 619 children reported that those exposed to preeclampsia had an increased risk of ADHD (aHR, 1.15; 95% CI, 1.12-1.19) compared with those not exposed to preeclampsia and SGA; after adjustment, the aHR for exposure to both preeclampsia and SGA was 1.43 (95% CI, 1.31-1.55), which was also comparable with that of exposure to SGA alone.^9^ In addition, a Norwegian population-based study^11^ including 980 560 children found that preeclampsia in term births was associated with increases in the risk of ADHD (adjusted odds ratio [AOR], 1.18; 95% CI, 1.05-1.33), ASD (AOR, 1.29; 95% CI, 1.08-1.54), and intellectual disability (AOR, 1.50; 95% CI, 1.13-1.97) after adjustment, but the researchers did not assess SGA exposure. A Swedish and Danish study^33^ of 4 489 044 births reported aHRs of 1.6 for both ASD and ADHD and 2.5 for intellectual disabilities among offspring exposed to preeclampsia who were born earlier than 33 weeks’ gestation; however, the aHR for exposure to preterm birth alone was not reported. Another Swedish and Danish study^12,13^ found a 2- to 3-fold increase in the risk of offspring psychosis or schizophrenia after preeclampsia exposure. Our study found associations with modest effect sizes that were consistent with those studies,^9,10,11,12,13,33^ but we also observed that the risk estimates for specific developmental disorders and ADHD and conduct disorders in offspring exposed to both preeclampsia and perinatal complications (ie, birth at <34 weeks’ gestation and/or SGA status) were higher (aHR, 2.82 for birth at <34 weeks’ gestation and 1.88 for SGA status) than those of offspring exposed to perinatal complications only, which is a novel finding.

Few studies have examined the association between exposure to preeclampsia and the risk of other neurodevelopmental and psychiatric disorders in offspring. A retrospective population-based cohort study^34^ in Israel including 253 808 singletons reported that exposure to preeclampsia was associated with obstructive sleep apnea, epilepsy, and cerebral palsy in offspring, whereas no association was found with eating disorders. Our study similarly detected no association between exposure to preeclampsia and the risk of eating disorders in offspring; however, we did find an association between preeclampsia alone and the risk of sleeping disorders, although this association was explained by familial confounding, and epilepsy and cerebral palsy were not assessed. Furthermore, we found that offspring exposed to both preeclampsia and perinatal complications had a higher likelihood of developing other behavioral and emotional disorders (*ICD-10* code F98), which was not explained by familial confounding; however, the effect size was similar to that of exposure to perinatal complications only. Notably, for intellectual disabilities and sleeping disorders, the effect size of exposure to both preeclampsia and perinatal complications was lower than that of exposure to perinatal complications only, and this lower effect size remained after adjusting for gestational age and birth weight (data not shown). Although the sample of offspring with sleeping disorders was small and the risk estimate for sleeping disorders was therefore less reliable, we could not explain the lower effect size for the risk of intellectual disabilities among those exposed to both preeclampsia and perinatal complications compared with those exposed to perinatal complications only.

To our knowledge, only3 population-based studies of ASD, ADHD, and intellectual disabilities conducted in Sweden and Denmark^9,10,33^ and 1 case control study of ASD conducted in Taiwan^35^ have assessed familial confounding by including sibling pairs who were discordant for preeclampsia, and no sibling-matched study has examined the associations between preeclampsia and other neuropsychiatric disorders. However, the findings of those studies^9,10,33,35^ suggested that familial confounding did not explain the associations between maternal preeclampsia and ASD, ADHD, and intellectual disabilities because no marked difference in effect size was observed between the whole population and the differentially exposed siblings. Notably, our sibling pair analysis did not detect true associations between preeclampsia alone and neurodevelopmental and psychiatric disorders because the associations were explained by unmeasured familial confounding. However, the associations of exposure to both preeclampsia and perinatal complications or exposure to perinatal complications alone with intellectual disabilities, specific developmental disorders, ADHD and conduct disorders, and other behavioral and emotional disorders (*ICD-10* code F98) were not explained by familial confounding. Further large population–based research is warranted to verify our findings.

The etiologic factors underlying preeclampsia are not well known. However, there are a few mechanisms that may explain the association between maternal preeclampsia and fetal neurodevelopment. First, the placental insufficiency associated with preeclampsia^36^ can lead to insufficient placental perfusion, hypoxia, and oxidative stress, which may have implications for neurodevelopment.^37,38,39,40,41^ Second, impaired balance between circulating proangiogenic and antiangiogenic factors from the placenta has been reported in mothers with preeclampsia, with possible consequences for fetal vascular development, which in turn could plausibly impact both fetal cerebrovascular function and neurodevelopment and be further associated with cognitive and developmental functions in postnatal life.^42^ Third, maternal inflammation may also play a mechanistic role.^43^ Fetal exposure to maternal allergies, autoimmune diseases, and infections has been reported to be associated with both preeclampsia and offspring neurodevelopment.^16,43^ In animal models, some maternal cytokines, such as C-reactive protein, interleukin 6, and interleukin 17, seem to be able to cross the placenta and enter fetal circulation, where they may regulate neuronal function and have consequences for later psychiatric and cognitive pathological characteristics.^44,45,46^

### Limitations

This study has several limitations. Although the study adjusted for several putative confounding factors and performed sibling pair analyses, unknown and unmeasured confounding of sibling-discordant factors remains a limitation. The study also lacks data on paternal factors. In addition, exploration of factors moderating and mediating the association of exposure to both preeclampsia and perinatal complications with neurodevelopmental and psychiatric disorders in offspring is warranted. Although we identified all recorded diagnoses of neuropsychiatric disorders in offspring until the oldest were age 22 years, changes in diagnosis and comorbidities were not taken into account. Given that the mean follow-up duration was 12.4 years, the rate of late-onset disorders was underestimated (eTable 1 in the Supplement).

## Conclusions

This cohort study found that offspring exposed to both maternal preeclampsia and perinatal complications had modestly increased risks of developing intellectual disabilities, specific developmental disorders, ADHD and conduct disorders, and other behavioral and emotional disorders. For specific developmental disorders and ADHD and conduct disorders, the risk estimates were higher among offspring exposed to both preeclampsia and perinatal complications compared with offspring exposed to perinatal complications only. Exposure to preeclampsia alone did not increase the risk of neurodevelopmental and psychiatric disorders because the detected associations were explainable by familial confounding. Further research is warranted to explore the complex mechanisms underlying the association between preeclampsia exposure and the development of these disorders.

## Notes

Supplement. **eMethods.** Data Sources **eTable 1.** *ICD-10* Codes and Corresponding Neuropsychiatric Disorders and Median Age at Onset of Diagnoses After Birth Until 2018 **eTable 2.** Numbers in Exposure Groups and Offspring Neurodevelopmental and Psychiatric Disorder Outcome Groups **eTable 3.** Number at Risk of Offspring Neuropsychiatric Disorders in Relation to Maternal Preeclampsia and Perinatal Complications **eTable 4.** Crude and Adjusted Hazard Ratios for Offspring Neuropsychiatric Disorders in Relation to Preeclampsia and Perinatal Complications **eTable 5.** Numbers in Exposure Groups and Offspring Psychotropic Medication Outcome Groups **eTable 6.** Sibling Pair Analysis in All First Pairs of Mothers With a Singleton Sibling Pair for Some Neurodevelopmental and Psychiatric Disorders in the Second Child **eTable 7.** Sibling Pair Analysis in All Mothers With a Singleton Sibling Pair (n = 438 626) Among the 1 012 723 Births (Born 1996-2014) for Psychotic Disorders, Anxiety Disorders, and Autism Spectrum Disorder in the Second Child **eTable 8.** Sibling Pair Analysis in All First Pairs of Mothers With a Singleton Sibling Pair (n = 438 626) Among the 1 012 723 Births (Born 1996-2014) for Psychotic Disorders, Anxiety Disorders, and Autism Spectrum Disorder in the Second Child **eTable 9.** Sibling Pair Analysis in All Mothers With a Singleton Sibling Pair (n = 427 591) Among the 1 012 723 Births (Born 1996-2014) for Any Psychiatric Disorder and Specific Neurodevelopmental Disorders in the Second Child **eTable 10.** Hazard Ratios for Offspring Neurodevelopmental and Psychiatric Disorders in Relation to Maternal Preeclampsia (*ICD-10* code O14) and Perinatal Complications **eTable 11.** Hazard Ratios for Offspring Psychotropic Medications in Relation to Maternal Preeclampsia (*ICD-10* code O14) and Perinatal Complications **eTable 12.** Hazard Ratios for Offspring Neurodevelopmental and Psychiatric Disorders in Relation to Maternal Preeclampsia and Perinatal Complications **eTable 13.** Hazard Ratios for Offspring Neurodevelopmental and Psychiatric Disorders in Relation to Gestational Hypertension and Perinatal Complications **eTable 14.** Mediating Effect of Perinatal Complications in the Association Between Maternal Preeclampsia and Any Offspring *ICD-10* F Diagnosis **eFigure 1.** Risks of Offspring Psychotropic Medication Purchase In Relation to Categories of Maternal Preeclampsia and Perinatal Complications **eFigure 2.** Mediating Effect of Perinatal Complications in the Association Between Maternal Preeclampsia and Any Offspring *ICD-10* F Diagnosis
