# Supplementary material for: Association of Preeclampsia and Perinatal Complications With Offspring Neurodevelopmental and Psychiatric Disorders
Source: JAMA Netw Open. 2022 Jan 28;5(1):e2145719. doi: 10.1001/jamanetworkopen.2021.45719 (PMC8800079; doi:10.1001/jamanetworkopen.2021.45719)
Supplement: Supplement. — eMethods. Data Sources eTable 1. ICD-10 Codes and Corresponding Neuropsychiatric Disorders and Median Age at Onset of Diagnoses After Birth Until 2018 eTable 2. Numbers in Exposure Groups and Offspring Neurodevelopmental and Psychiatric Disorder Outcome Groups eTable 3. Number at Risk of Offspring Neuropsychiatric Disorders in Relation to Maternal Preeclampsia and Perinatal Complications eTable 4. Crude and Adjusted Hazard Ratios for Offspring Neuropsychiatric Disorders in Relation to Preeclampsia and Perinatal Complications eTable 5. Numbers in Exposure Groups and Offspring Psychotropic Medication Outcome Groups eTable 6. Sibling Pair Analysis in All First Pairs of Mothers With a Singleton Sibling Pair for Some Neurodevelopmental and Psychiatric Disorders in the Second Child eTable 7. Sibling Pair Analysis in All Mothers With a Singleton Sibling Pair (n = 438 626) Among the 1 012 723 Births (Born 1996-2014) for Psychotic Disorders, Anxiety Disorders, and Autism Spectrum Disorder in the Second Child eTable 8. Sibling Pair Analysis in All First Pairs of Mothers With a Singleton Sibling Pair (n = 438 626) Among the 1 012 723 Births (Born 1996-2014) for Psychotic Disorders, Anxiety Disorders, and Autism Spectrum Disorder in the Second Child eTable 9. Sibling Pair Analysis in All Mothers With a Singleton Sibling Pair (n = 427 591) Among the 1 012 723 Births (Born 1996-2014) for Any Psychiatric Disorder and Specific Neurodevelopmental Disorders in the Second Child eTable 10. Hazard Ratios for Offspring Neurodevelopmental and Psychiatric Disorders in Relation to Maternal Preeclampsia (ICD-10 code O14) and Perinatal Complications eTable 11. Hazard Ratios for Offspring Psychotropic Medications in Relation to Maternal Preeclampsia (ICD-10 code O14) and Perinatal Complications eTable 12. Hazard Ratios for Offspring Neurodevelopmental and Psychiatric Disorders in Relation to Maternal Preeclampsia and Perinatal Complications eTable 13. Hazard Ratios for Offspring Neurodevelopme [file jamanetwopen-e2145719-s001.pdf]

Supplementary Online Content

Kong L, Chen X, Liang Y, Forsell Y, Gissler M, Lavebratt C. Association of preeclampsia and perinatal complications with offspring neurodevelopmental and psychiatric disorders. *JAMA Netw Open*. 2022;5(1):e2145719. doi:10.1001/jamanetworkopen.2021.45719

eMethods. Data Sources

eTable 1. *ICD-10* Codes and Corresponding Neuropsychiatric Disorders and Median Age at Onset of Diagnoses After Birth Until 2018

eTable 2. Numbers in Exposure Groups and Offspring Neurodevelopmental and Psychiatric Disorder Outcome Groups

eTable 3. Number at Risk of Offspring Neuropsychiatric Disorders in Relation to Maternal Preeclampsia and Perinatal Complications

eTable 4. Crude and Adjusted Hazard Ratios for Offspring Neuropsychiatric Disorders in Relation to Preeclampsia and Perinatal Complications

eTable 5. Numbers in Exposure Groups and Offspring Psychotropic Medication Outcome Groups

eTable 6. Sibling Pair Analysis in All First Pairs of Mothers With a Singleton Sibling Pair for Some Neurodevelopmental and Psychiatric Disorders in the Second Child

eTable 7. Sibling Pair Analysis in All Mothers With a Singleton Sibling Pair (n = 438 626) Among the 1 012 723 Births (Born 1996-2014) for Psychotic Disorders, Anxiety Disorders, and Autism Spectrum Disorder in the Second Child

eTable 8. Sibling Pair Analysis in All First Pairs of Mothers With a Singleton Sibling Pair (n = 300 247) Among the 1 012 723 Births (Born 1996-2014) for Psychotic Disorders, Anxiety Disorders, and Autism Spectrum Disorder in the Second Child

eTable 9. Sibling Pair Analysis in All Mothers With a Singleton Sibling Pair (n = 427 591) Among the 1 012 723 Births (Born 1996-2014) for Any Psychiatric Disorder and Specific Neurodevelopmental Disorders in the Second Child

eTable 10. Hazard Ratios for Offspring Neurodevelopmental and Psychiatric Disorders in Relation to Maternal Preeclampsia (*ICD-10* code O14) and Perinatal Complications

eTable 11. Hazard Ratios for Offspring Psychotropic Medications in Relation to Maternal Preeclampsia (*ICD-10* code O14) and Perinatal Complications

eTable 12. Hazard Ratios for Offspring Neurodevelopmental and Psychiatric Disorders in Relation to Maternal Preeclampsia and Perinatal Complications

eTable 13. Hazard Ratios for Offspring Neurodevelopmental and Psychiatric Disorders in Relation to Gestational Hypertension and Perinatal Complications

eTable 14. Mediating Effect of Perinatal Complications in the Association Between Maternal Preeclampsia and Any Offspring *ICD-10* F Diagnosis

eFigure 1. Risks of Offspring Psychotropic Medication Purchase In Relation to Categories of Maternal Preeclampsia and Perinatal Complications

eFigure 2. Mediating Effect of Perinatal Complications in the Association Between Maternal Preeclampsia and Any Offspring *ICD-10* F Diagnosis

This supplementary material has been provided by the authors to give readers additional information about their work.

© 2022 Kong L et al. *JAMA Network Open*.

eMethods. Data Sources

MBR includes data on all live births and stillbirths in Finland since 1987 with a gestational age of ≥ 22 weeks or a birth weight ≥ 500 g. The Finnish Register on Reimbursement Drugs (RRD) is kept by the Finnish Social Insurance Institution (SII). Since 1994, RRD has registered all reimbursed drug prescriptions (ATC-code) that were dispensed at pharmacies. The Finnish Care Registers for Health Care (HILMO) contains data on all hospital in-patient treatments (since 1969) as well as out-patient treatments by physicians in specialized care (since 1998), and covers adult psychiatric diagnoses well according to validation studies,[1] while there is only one validation study on pediatric psychiatric diagnoses in HILMO, and that one reported good validity for pediatric autism spectrum disorder.[2] Information from the different registers and datasets were linked and merged using personal identification numbers (PIN) assigned to all Finnish citizens and permanent residents.

References

1. Sund R. Quality of the Finnish Hospital Discharge Register: a systematic review. Scand J Public Health 2012; 40(6):505-515.
2. Lampi K, Sourander A, Gissler M, et al. Brief report: validity of Finnish registry-based diagnoses of autism with the ADI-R. Acta Paediatr 2010; 99(9):1425-1428.

© 2022 Kong L et al. *JAMA Network Open*.

eTable 1. *ICD-10* Codes and Corresponding Neuropsychiatric Disorders and Median Age at Onset of Diagnoses After Birth Until 2018

Median with 25th and 75th percentiles.

**ICD-10 codes**	**Offspring disorders**	**Proportion identified of the cases predicted to be diagnosed before 22 years of age, %[a]**	**Age at onset. Median (year)**	
			**Birth cohort 1996-2014**	
			Median	25th-75th percentiles
F20-29	Psychotic disorders	30.3	14.1	9.0-16.7
F30-39, F92	Mood disorders	46.5	13.6	9.0-16.0
F40-43, F93	Anxiety disorders	55.5	12.4	7.6-15.5
F50	Eating disorders	43.7	14.2	11.5-15,8
F51	Sleeping disorders	61.3	1.9	0.8-9.9
F60-69	Personality disorders	34.6	14.2	8.4-16.9
F70-79	Intellectual disabilities	83.2	3.8	2.6-5.5
F80-83	Specific developmental disorders	93.8	5.3	3.8-7.3
F84	Autism spectrum disorder (ASD)	86.2	5.8	3.6-8.9
F90–91	Attention deficit hyperactivity disorder (ADHD) and conduct disorders	88.4	7.8	5.7-10.5
F98	Other behavioral and emotional disorders	100.0	6.1	4.1-8.5

Abbreviations: ICD-10, International Statistical Classification of Diseases and Related Health Problems, 10th Revision. IQR, interquartile range. ASD, Autism spectrum disorder. ADHD, attention deficit/hyperactivity disorder.
[a]Proportion identified of the number of cases predicted to be diagnosed before 22 years of age. This was estimated as number of children diagnosed latest 2018 in the 1996-2014 birth cohort divided by the following: the proportion of children with diagnosis in the 1996 birth cohort, times (*), the total number of children in the 1996-2014 birth cohort.

© 2022 Kong L et al. *JAMA Network Open*.

eTable 2. Numbers in Exposure Groups and Offspring Neurodevelopmental and Psychiatric Disorder Outcome Groups

1 012 723 singleton live births 1996–2014 in Finland followed until 2018.

Exposures[a]	No. (%)											
	Any F-diagnosis (n=93 281)	Psychotic disorders F20-F29 (n=2904)	Mood disorders F30-39, F92 (n=38 293)	Anxiety disorders F40-43, F93 (n=50 731)	Eating disorders F50 (n=6 029)	Sleeping disorders F51 (n=6 153)	Personality disorders F60-69 (n=3 195)	Intellectual disabilities F70-79 (n=8 014)	Specific developmental disorders F80-83 (n=55 326)	Autism spectrum disorders F84 (n=10 513)	ADHD& conduct disorders F90–91 (n=30 115)	Other behavioral& emotional disorders F98 (n=27 092)
No preeclampsia, no perinatal complications (N=953 197)	85 147 (8.9)	2 708 (0.3)	35 763 (3.8)	47 259 (5.0)	5 615 (0.6)	5 729 (0.6)	2 942 (0.3)	6 747 (0.7)	49 442 (5.2)	9 548 (1.0)	27 461 (2.9)	24 643 (2.6)
Preeclampsia without perinatal complications (N=21 030)	2 014 (9.6)	45 (0.2)	733 (3.5)	1 048 (5.0)	118 (0.6)	145 (0.7)	57 (0.3)	175 (0.8)	1 357 (6.5)	286 (1.4)	744 (3.5)	645 (3.1)
Preeclampsia with perinatal complications (N=4 891)	798 (16.3)	18 (0.4)	176 (3.6)	266 (5.4)	35 (0.7)	20 (0.4)	20 (0.4)	104 (2.1)	638 (13.0)	87 (1.8)	239 (4.9)	242 (4.9)
Perinatal complications, but no preeclampsia (N=33 625)	5 322 (15.8)	133 (0.4)	1 621 (4.8)	2 158 (6.4)	261 (0.8)	259 (0.8)	176 (0.5)	988 (2.9)	3 889 (11.6)	592 (1.8)	1 671 (5.0)	1 562 (4.6)

[a] Preeclampsia was ICD-10 codes O11 or O14. Perinatal complications referred to SGA or delivery before 34 gestational weeks. The no preeclampsia, no perinatal complications group excluded maternal chronic hypertension and gestational hypertension.

© 2022 Kong L et al. *JAMA Network Open*.

eTable 3. Number at Risk of Offspring Neuropsychiatric Disorders in Relation to Maternal Preeclampsia and Perinatal Complications

1 012 723 singleton live births 1996–2014 in Finland followed until 2018.

**Exposures**[a]	**Age in years**																					
	0	1	2	3	4	5	6	7	8	9	10	11	12	13	14	15	16	17	18	19	20	21
**Specific developmental disorders (F80-83)**																						
No preeclampsia	0	1352	1121	2750	8025	8098	8182	5935	3292	2494	2116	1464	1171	913	711	629	565	272	133	43	22	13
Perinatal complications only	0	306	233	338	593	573	612	405	224	141	109	93	51	58	31	27	25	15	8	3	0	1
Preeclampsia without perinatal complications	0	43	31	97	224	245	223	168	77	70	42	37	27	18	15	13	11	7	4	0	1	0
Preeclampsia with perinatal complications	0	63	43	71	101	87	101	67	24	18	18	11	12	5	3	8	3	2	1	1	0	0
**ADHD and conduct disorders (F90-91)**																						
No preeclampsia	0	409	220	493	1392	1990	2993	3221	3359	3028	2401	1780	1358	1081	1106	1052	680	420	314	128	84	60
Perinatal complications only	0	68	45	80	123	153	206	187	173	173	114	88	67	49	39	31	27	11	14	4	4	2
Preeclampsia without perinatal complications	0	20	3	15	38	74	96	97	96	77	69	45	37	27	18	18	11	5	3	2	1	0
Preeclampsia with perinatal complications	0	16	3	21	25	24	32	30	28	16	15	9	7	5	2	3	2	3	1	1	0	0

[a] No preeclampsia referred to no preeclampsia, no SGA, and born ≥34 weeks of gestation after excluding maternal chronic hypertension and gestational hypertension.

© 2022 Kong L et al. *JAMA Network Open*.

eTable 4. Crude and Adjusted Hazard Ratios for Offspring Neuropsychiatric Disorders in Relation to Preeclampsia and Perinatal Complications (1 012 723 Singleton Live Births 1996–2014 in Finland Followed Up Until 2018)

Corresponds to Figure 2.

**Exposures**[a]	Any F-diagnosis (n=93 281)	Psychotic disorders F20-F29 (n=2904)	Mood disorders F30-39, F92 (n=38 293)	Anxiety disorders F40-43, F93 (n=50 731)	Eating disorders F50 (n=6 029)	Sleeping disorders F51 (n=6 153)	Personality disorders F60-69 (n=3 195)	Intellectual disabilities F70-79 (n=8 014)	Specific development al disorders F80-83 (n=55 326)	Autism spectrum disorders F84 (n=10 513)	ADHD& conduct disorders F90–91 (n=30 115)	Other behavioral& emotional disorders F98 (n=27 092)
**Crude model (Crude HR, 95% CI)**												
No preeclampsia	1.00	1.00	1.00	1.00	1.00	1.00	1.00	1.00	1.00	1.00	1.00	1.00
Preeclampsia without perinatal complications	1.14 (1.09-1.19)	0.91 (0.69-1.20)	1.12 (1.04-1.20)	1.18 (1.11-1.26)	1.16 (0.97-1.39)	1.22 (1.04-1.44)	1.08 (0.83-1.40)	1.20 (1.03-1.40)	1.29 (1.22-1.36)	1.43 (1.27-1.60)	1.32 (1.22-1.42)	1.24 (1.15-1.34)
Preeclampsia with perinatal complications	2.05 (1.91-2.20)	1.58 (1.02-2.45)	1.19 (1.03-1.38)	1.34 (1.19-1.51)	1.52 (1.09-2.12)	0.74 (0.48-1.15)	1.65 (1.07-2.57)	3.21 (2.64-3.89)	2.75 (2.55-2.98)	1.97 (1.59-2.43)	1.94 (1.70-2.20)	2.11 (1.86-2.40)
Perinatal complications only	1.91 (1.86-1.96)	1.61 (1.37-1.90)	1.44 (1.37-1.51)	1.44 (1.38-1.50)	1.47 (1.30-1.67)	1.35 (1.19-1.53)	1.89 (1.62-2.20)	4.36 (4.08-4.67)	2.38 (2.31-2.46)	1.89 (1.74-2.05)	1.88 (1.79-1.98)	1.93 (1.83-2.03)
**Adjusted model**[b] **(Adjusted HR, 95% CI)**												
No preeclampsia	1.00	1.00	1.00	1.00	1.00	1.00	1.00	1.00	1.00	1.00	1.00	1.00
Preeclampsia without perinatal complications	1.18 (1.12-1.23)	0.97 (0.73-1.28)	1.10 (1.02-1.18)	1.13 (1.06-1.20)	1.13 (0.94-1.36)	1.18 (1.00-1.40)	1.12 (0.86-1.46)	1.22 (1.05-1.42)	1.24 (1.18-1.31)	1.23 (1.09-1.39)	1.22 (1.13-1.31)	1.14 (1.06-1.24)
Preeclampsia with perinatal complications	2.11 (1.96-2.26)	1.60 (1.03-2.49)	1.18 (1.02-1.37)	1.28 (1.14-1.45)	1.40 (0.99-1.95)	0.64 (0.40-1.02)	1.54 (0.98-2.42)	3.34 (2.75-4.06)	2.82 (2.60-3.05)	1.73 (1.40-2.13)	1.88 (1.65-2.14)	2.04 (1.80-2.32)
Perinatal complications only	1.77 (1.72-1.82)	1.40 (1.19-1.65)	1.28 (1.22-1.34)	1.28 (1.23-1.34)	1.44 (1.27-1.63)	1.22 (1.07-1.38)	1.64 (1.41-1.92)	4.22 (3.95-4.52)	2.26 (2.18-2.33)	1.67 (1.53-1.81)	1.60 (1.52-1.68)	1.81 (1.71-1.90)

[a] Exclusion: Births with maternal in-hospital psychiatric disorder and those with maternal pre-gestational diabetes. Preeclampsia was ICD-10 codes O11 or O14. Perinatal complications referred to SGA or delivery before 34 gestational weeks. The no preeclampsia, no perinatal complications group excluded maternal chronic hypertension and gestational hypertension.

[b] The analyses were adjusted for offspring birth year, sex, maternal age at delivery, mother's country of birth (Finland or not), mother married at birth (yes/no), maternal occupation (SES, upper white collar worker, lower white collar worker, blue collar worker, and other status), maternal smoking (yes/no), parity (0 or ≥1), maternal obesity (ICD-10: E65-E66, yes/no), maternal gestational diabetes (yes/no), maternal outpatient psychiatric disorders (yes/no), maternal N05/N06 purchase during pregnancy, and maternal systemic inflammatory disease (yes/no).

© 2022 Kong L et al. *JAMA Network Open*.

eTable 5. Numbers in Exposure Groups and Offspring Psychotropic Medication Outcome Groups

1 012 723 singleton live births 1996–2014 in Finland followed until 2018.

	**No. (%)**			
**Exposures[a]**	Any medication (N=50 131)	Anxiolytics, hypnotics and sedatives (N=33 471)	Antidepressants (N=11 509)	Stimulants (N=14 547)
No preeclampsia, no perinatal complications (N=953 197)	46 463 (4.9)	31 060 (3.3)	10 809 (1.1)	13 283 (1.4)
Preeclampsia without perinatal complications (N=21 030)	931 (4.4)	592 (2.8)	174 (0.8)	332 (1.6)
Preeclampsia with perinatal complications (N=4 891)	293 (6.0)	198 (4.0)	55 (1.1)	94 (1.9)
Perinatal complications, no preeclampsia (N=33 625)	2 444 (7.3)	1 621 (4.8)	471 (1.4)	838 (2.5)

[a] Preeclampsia was ICD-10 codes O11 or O14. Perinatal complications referred to SGA or delivery before 34 gestational weeks. The no preeclampsia, no perinatal complications group excluded maternal chronic hypertension and gestational hypertension.

© 2022 Kong L et al. *JAMA Network Open*.

eTable 6. Sibling Pair Analysis in All First Pairs[a] of Mothers With a Singleton Sibling Pair (n = 300 247) Among the 1 012 723 Births (Born 1996-2014) for Some Neurodevelopmental and Psychiatric Disorders in the Second Child

Adjusted Cox hazard ratios (HRs) and 95% CIs for the outcomes (columns) in the second child being some neurodevelopmental and psychiatric disorders from birth until 2018, in relation to exposure for the sibling pair to preeclampsia with perinatal complications.

**Exposure to preeclampsia with perinatal complications[b]**	**Adjusted HR (95% CI)**				
	Any F-diagnosis	Intellectual disabilities (F70-79)	Specific developmental disorders (F80-83)	ADHD and conduct disorders (F90-91)	Other behavioral and emotional disorders (F98)
**Model 1[c]**					
None of the siblings in the pair was exposed (n=298 066)	1.00	1.00	1.00	1.00	
Exposure in the first, not in the second pregnancy/child (n=1464)	0.93（0.75-1.14）	0.60（0.27-1.33）	0.99（0.78-1.25）	0.92（0.65-1.31）	1.00（0.72-1.3）
Exposure in the second, not in the first pregnancy/child (n=597)	2.05（1.64-2.57）	2.52（1.35-4.69）	3.04（2.44-3.78）	1.77（1.15-2.71）	1.17（0.70-1.94）
Both of the siblings in the pair were exposed (n=120)	3.00（1.90-4.66）	3.98（1.28-12.36）	3.57（2.25-5.66）	2.36（0.98-5.67）	1.61（0.63-4.29）
**Model 2[d]**					
None of the siblings in the pair was exposed (n=298 066)	1.00	1.00	1.00	1.00	
Exposure in the first, not in the second pregnancy/child (n=1464)	0.86（0.70-1.06）	0.55（0.25-1.24）	0.92（0.73-1.16）	0.85（0.60-1.21）	0.95（0.68-1.32）
Exposure in the second, not in the first pregnancy/child (n=597)	2.01（1.60-2.51）	2.45（1.31-4.55）	2.95（2.37-3.67）	1.72（1.12-2.64）	1.15（0.69-1.91）
Both of the siblings in the pair were exposed (n=120)	2.92（1.86-4.57）	3.88（1.25-12.04）	3.49（2.20-5.53）	2.33（0.97-5.60）	1.58（0.60-4.23）

[a] First sibling pairs were included, i.e. sibling 1&2.
[b] Exclusion: Maternal in-hospital psychiatric disorder and maternal pre-gestational diabetes. Reference group was no preeclampsia (no ICD-10 O11 or O14) and no perinatal complications after excluding maternal chronic hypertension and gestational hypertension.
[c] Model 1: The analyses were adjusted for offspring birth year, sex, maternal age at delivery, mother's country of birth (Finland or not), mother married at birth (yes/no), maternal occupation (SES, upper white collar worker, lower white collar worker, blue collar worker, and other status), maternal smoking (yes/no), parity (0 or ≥1), maternal obesity (ICD-10: E65-E66, yes/no), maternal gestational diabetes (yes/no), maternal outpatient psychiatric disorders (yes/no), maternal N05/N06 purchase during pregnancy (yes/no), maternal systemic inflammatory disease (yes/no), and intra-pregnancy interval.
[d] Model 2: Adjusted for the variables in Model 1 and presence of the studied F diagnosis or psychotropic medication in the first child.

© 2022 Kong L et al. *JAMA Network Open*.

eTable 7. Sibling Pair Analysis in All Mothers With a Singleton Sibling Pair[a] (n = 438 626) Among the 1 012 723 Births (Born 1996-2014) for Psychotic Disorders, Anxiety Disorders, and Autism Spectrum Disorder in the Second Child

Adjusted Cox hazard ratios (HRs) and 95% CIs for the outcomes (columns) in the second child being (i) Psychotic disorders (F20-29), (ii) Anxiety disorders (F40-43, F93), and (iii) ASD (F84) from birth until 2018, in relation to exposure for the sibling pair to preeclampsia with perinatal complications.

**Exposure to preeclampsia with perinatal complications[b]**	**Adjusted HR (95% CI)**		
	Psychotic disorders (F20-29)	Anxiety disorders (F40-43, F93)	ASD (F84)
**Model 1[c]**			
None of the siblings in the pair was exposed (n=435 997)	1.00	1.00	1.00
Exposure in the first, not in the second pregnancy/child (n=1614)	NA	1.27 (0.95-1.68)	1.01 (0.57-1.78)
Exposure in the second, not in the first pregnancy/child (n=873)	1.24 (0.17-8.82)	1.22 (0.79-1.89)	1.60 (0.83-3.08)
Both of the siblings in the pair were exposed (n=142)	NA	0.51 (0.07-3.61)	NA
**Model 2[d]**			
None of the siblings in the pair was exposed (n=435 997)	1.00	1.00	1.00
Exposure in the first, not in the second pregnancy/child (n=1614)	NA	1.17 (0.88-1.55)	0.93 (0.53-1.64)
Exposure in the second, not in the first pregnancy/child (n=873)	1.20 (0.17-8.55)	1.20 (0.77-1.86)	1.54 (0.80-2.97)
Both of the siblings in the pair were exposed (n=142)	NA	0.48 (0.07-3.44)	NA

NA, not applicable.
[a] All consecutive sibling pairs were included, i.e. sibling 1&2, 2&3 but not 1&3.
[b] Exclusion: Maternal in-hospital psychiatric disorder and maternal pre-gestational diabetes. Reference group was no preeclampsia (no ICD-10 O11 or O14) and no perinatal complications after excluding maternal chronic hypertension and gestational hypertension.
[c] Model 1: The analyses were adjusted for offspring birth year, sex, maternal age at delivery, mother's country of birth (Finland or not), mother married at birth (yes/no), maternal occupation (SES, upper white collar worker, lower white collar worker, blue collar worker, and other status), maternal smoking (yes/no), parity (0 or ≥1), maternal obesity (ICD-10: E65-E66, yes/no), maternal gestational diabetes (yes/no), maternal outpatient psychiatric disorders (yes/no), maternal N05/N06 purchase during pregnancy (yes/no), maternal systemic inflammatory disease (yes/no), and intra-pregnancy interval.
[d] Model 2: Adjusted for the variables in Model 1 and presence of the studied F diagnosis in the first child.

© 2022 Kong L et al. *JAMA Network Open*.

eTable 8. Sibling Pair Analysis in All First Pairs[a] of Mothers With a Singleton Sibling Pair (n = 300 247) Among the 1 012 723 Births (Born 1996-2014) for Psychotic Disorders, Anxiety Disorders, and Autism Spectrum Disorder in the Second Child

Adjusted Cox hazard ratios (HRs) and 95% CIs for the outcomes (columns) in the second child being (i) Psychotic disorders (F20-29), (ii) Anxiety disorders (F40-43, F93), and (iii) ASD (F84) from birth until 2018, in relation to exposure for the sibling pair to preeclampsia with perinatal complications.

**Exposure to preeclampsia with perinatal complications[b]**	**Adjusted HR (95% CI)**		
	Psychotic disorders (F20-29)	Anxiety disorders (F40-43, F93)	ASD (F84)
**Model 1[c]**			
None of the siblings in the pair was exposed (n=298 066)	1.00	1.00	1.00
Exposure in the first, not in the second pregnancy/child (n=1464)	NA	1.20 (0.89-1.62)	0.90 (0.48-1.67)
Exposure in the second, not in the first pregnancy/child (n=597)	1.42 (0.20-10.11)	1.18 (0.73-1.93)	2.14 (1.11-4.13)
Both of the siblings in the pair were exposed (n=120)	NA	0.55 (0.08-3.89)	NA
**Model 2[d]**			
None of the siblings in the pair was exposed (n=298 066)	1.00	1.00	1.00
Exposure in the first, not in the second pregnancy/child (n=1464)	NA	1.11 (0.83-1.50)	0.82 (0.44-1.53)
Exposure in the second, not in the first pregnancy/child (n=597)	1.38 (0.19-9.82)	1.17 (0.72-1.91)	2.08 (1.08-4.01)
Both of the siblings in the pair were exposed (n=120)	NA	0.54 (0.08-3.81)	NA

NA, not applicable.
[a] First sibling pairs were included, i.e. sibling 1&2.
[b] Exclusion: Maternal in-hospital psychiatric disorder and maternal pre-gestational diabetes. Reference group was no preeclampsia (no ICD-10 O11 or O14) and no perinatal complications after excluding maternal chronic hypertension and gestational hypertension.
[c] Model 1: The analyses were adjusted for offspring birth year, sex, maternal age at delivery, mother’s country of birth (Finland or not), mother married at birth (yes/no), maternal occupation (SES, upper white collar worker, lower white collar worker, blue collar worker, and other status), maternal smoking (yes/no), parity (0 or ≥1), maternal obesity (ICD-10: E65-E66, yes/no), maternal gestational diabetes (yes/no), maternal outpatient psychiatric disorders (yes/no), maternal N05/N06 purchase during pregnancy (yes/no), maternal systemic inflammatory disease (yes/no), and intra-pregnancy interval.
[d] Model 2: Adjusted for the variables in Model 1 and presence of the studied F diagnosis in the first child.

© 2022 Kong L et al. *JAMA Network Open*.

eTable 9. Sibling Pair Analysis in All Mothers With a Singleton Sibling Pair (n = 427 591) Among the 1 012 723 Births (Born 1996-2014) for Any Psychiatric Disorder and Specific Neurodevelopmental Disorders in the Second Child

Adjusted Cox hazard ratios (HRs) and 95% CIs for the outcomes (columns) in the second child being (i) any psychiatric disorder (ICD-10 F diagnoses), and (ii) specific developmental disorders (F80-83) from birth until 2018, in relation to exposure for the sibling pair to preeclampsia.

**Exposure to preeclampsia (ICD-10: O11 or O14)**[a]	**Adjusted HR (95% CI)**	
	Any F-diagnosis	Specific developmental disorders (F80-83)
**Model 1**[b]		
None of the siblings in the pair was exposed (n=415 393)	1.00	1.00
Exposure in the first, not in the second pregnancy/child (n=6 636)	1.17 (1.06-1.28)	1.13 (1.01-1.26)
Exposure in the second, not in the first pregnancy/child (n=3 576)	1.18 (1.05-1.33)	1.23 (1.07-1.40)
Both of the siblings in the pair were exposed (n=1 986)	1.16 (0.99-1.36)	1.26 (1.05-1.51)
**Model 2**[c]		
None of the siblings in the pair was exposed (n=415 393)	1.00	1.00
Exposure in the first, not in the second pregnancy/child (n=6 636)	1.15 (1.04-1.26)	1.11 (1.00-1.23)
Exposure in the second, not in the first pregnancy/child (n=3 576)	1.16 (1.03-1.31)	1.20 (1.05-1.37)
Both of the siblings in the pair were exposed (n=1 986)	1.15 (0.98-1.35)	1.26 (1.05-1.51)

[a] Exclusion: Births with maternal in-hospital psychiatric disorder, maternal pre-gestational diabetes and perinatal complications.
[b] Model 1: The analyses were adjusted for offspring birth year, sex, maternal age at delivery, mother's country of birth (Finland or not), mother married at birth (yes/no), maternal occupation (SES, upper white collar worker, lower white collar worker, blue collar worker, and other status), maternal smoking (yes/no), parity (0 or ≥1), maternal obesity (ICD-10: E65-E66, yes/no), maternal gestational diabetes (yes/no), maternal outpatient psychiatric disorders (yes/no), maternal N05/N06 purchase during pregnancy (yes/no), maternal systemic inflammatory disease (yes/no), and intra-pregnancy interval.
[c] Model 2: Adjusted for the variables in Model 1 and presence of the studied F diagnosis or psychotropic medication in the first child.

© 2022 Kong L et al. *JAMA Network Open*.

eTable 10. Hazard Ratios for Offspring Neurodevelopmental and Psychiatric Disorders in Relation to Maternal Preeclampsia (*ICD-10* code O14) and Perinatal Complications

Maternal preeclampsia (PE) (*ICD-10* code O14) and perinatal complications (SGA or delivery before 34 gestational weeks) (1 012 723 singleton live births 1996–2014 in Finland followed until 2018).

PE stratified by SGA and gestational age[a]	Any F-diagnosis (n=93 281)	Psychotic disorders F20-F29 (n=2904)	Mood disorders F30-39, F92 (n=38 293)	Anxiety disorders F40-43, F93 (n=50 731)	Eating disorders F50 (n=6 029)	Sleeping disorders F51 (n=6 153)	Personality disorders F60-69 (n=3 195)	Intellectual disabilities F70-79 (n=8 014)	Specific develop-mental disorders F80-83 (n=55 326)	Autism spectrum disorders F84 (n=10 513)	ADHD& conduct disorders F90–91 (n=30 115)	Other behavioral& emotional disorders F98 (n=27 092)
**Crude model (Crude HR, 95% CI)**												
No PE no perinatal complications	1.00	1.00	1.00	1.00	1.00	1.00	1.00	1.00	1.00	1.00	1.00	1.00
PE without perinatal complications	0.80 (0.73-0.88)	1.61 (1.36-1.89)	0.88 (0.72-1.07)	1.09 (0.95-1.25)	1.03 (0.62-1.72)	0.99 (0.73-1.35)	0.22 (0.06-0.88)	0.89 (0.67-1.17)	1.23 (1.12-1.34)	1.29 (1.05-1.59)	1.37 (1.21-1.55)	1.18 (1.03-1.35)
PE with perinatal complications	1.74 (1.54-1.97)	0.36 (0.12-1.12)	0.53 (0.32-0.89)	1.38 (1.07-1.76)	1.97 (0.94-4.14)	0.28 (0.09-0.87)	0.88 (0.22-3.53)	2.60 (1.90-3.56)	2.81 (2.50-3.16)	2.12 (1.54-2.91)	2.03 (1.65-2.50)	2.55 (2.13-3.05)
Perinatal complications only	1.93 (1.88-1.98)	0.98 (0.24-3.90)	1.42 (1.36-1.49)	1.42 (1.37-1.49)	1.47 (1.30-1.65)	1.32 (1.17-1.49)	1.88 (1.62-2.17)	4.29 (4.02-4.58)	2.40 (2.32-2.47)	1.87 (1.73-2.03)	1.87 (1.79-1.97)	1.91 (1.82-2.00)
**Adjusted model [b] (Adjusted HR, 95% CI)**												
No PE no perinatal complications	1.00	1.00	1.00	1.00	1.00	1.00	1.00	1.00	1.00	1.00	1.00	1.00
PE without perinatal complications	1.09 (0.99-1.20)	1.44 (1.23-1.70)	0.96 (0.79-1.16)	1.02 (0.88-1.17)	1.21 (0.73-2.01)	0.99 (0.72-1.35)	0.41 (0.10-1.66)	1.01 (0.77-1.33)	1.14 (1.04-1.25)	1.11 (0.90-1.37)	1.14 (1.01-1.29)	1.06 (0.93-1.21)
PE with perinatal complications	2.51 (2.22-2.84)	0.93 (0.30-2.89)	0.62 (0.37-1.02)	1.32 (1.03-1.69)	2.23 (1.06-4.68)	0.28 (0.09-0.88)	1.71 (0.43-6.84)	3.07 (2.24-4.21)	2.75 (2.45-3.10)	1.87 (1.36-2.58)	1.86 (1.51-2.29)	2.41 (2.01-2.88)
Perinatal complications only	1.78 (1.73-1.82)	2.66 (0.66-10.67)	1.28 (1.22-1.34)	1.28 (1.23-1.33)	1.42 (1.26-1.60)	1.20 (1.06-1.35)	1.64 (1.42-1.90)	4.16 (3.90-4.45)	2.28 (2.21-2.36)	1.65 (1.52-1.78)	1.62 (1.55-1.70)	1.80 (1.71-1.89)

Preeclampsia was ICD-10 code: O14. Perinatal complications referred to SGA or delivery before 34 gestational weeks.
Exclusion: Maternal in-hospital psychiatric disorder and maternal pre-gestational diabetes.
[a] Reference group was no PE, no SGA, and born ≥34 weeks of gestation after excluding maternal chronic hypertension and gestational hypertension.
[b] The analyses were adjusted for offspring birth year, sex, maternal age at delivery, mother's country of birth (Finland or not), mother married at birth (yes/no), maternal occupation (SES, upper white collar worker, lower white collar worker, blue collar worker, and other status), maternal smoking (yes/no), parity (0 or ≥1), maternal obesity (ICD-10: E65-E66, yes/no), maternal gestational diabetes (yes/no), maternal outpatient psychiatric disorders (yes/no), maternal N05/N06 purchase during pregnancy, and maternal systemic inflammatory disease (yes/no).

© 2022 Kong L et al. *JAMA Network Open*.

eTable 11. Hazard Ratios for Offspring Psychotropic Medications in Relation to Maternal Preeclampsia (*ICD-10* code O14) and Perinatal Complications

Maternal preeclampsia (PE) (*ICD-10* code O14) and perinatal complications (SGA or delivery before 34 gestational weeks) (1 012 723 singleton live births 1996–2014 in Finland followed up until 2018).

PE stratified by SGA and gestational age[a]	Any medication (N=50 131)	Anxiolytics, hypnotics and sedatives (N=33 471)	Antidepressants (N=11 509)	Stimulants (N=14 547)
**Crude model (crude HR, 95% CI)**				
No PE no perinatal complications	1.00	1.00	1.00	1.00
PE without perinatal complications	1.19 (1.03-1.36)	1.26 (1.06-1.50)	0.11 (0.04-0.33)	1.09 (0.88-1.35)
PE with perinatal complications	1.58 (1.24-2.01)	1.87 (1.41-2.48)	0.15 (0.02-1.04)	1.10 (0.72-1.69)
Perinatal complications only	1.65 (1.58-1.71)	1.64 (1.56-1.72)	1.39 (1.28-1.52)	1.94 (1.82-2.08)
**Adjusted model[b] (adjusted HR, 95% CI)**				
No PE no perinatal complications	1.00	1.00	1.00	1.00
PE without perinatal complications	1.18 (1.02-1.35)	1.09 (0.91-1.29)	0.67 (0.22-2.07)	1.34 (1.08-1.67)
PE with perinatal complications	1.65 (1.30-2.10)	1.66 (1.26-2.20)	0.99 (0.14-7.05)	1.54 (1.00-2.36)
Perinatal complications only	1.53 (1.47-1.59)	1.54 (1.47-1.62)	1.26 (1.15-1.37)	1.68(1.57-1.80)

Abbreviations: HR, hazard ratios; PE, pre-eclampsia; SGA, small for gestational age.
Preeclampsia was ICD-10 code: O14. Perinatal complications referred to SGA or delivery before 34 gestational weeks.
Exclusion: Maternal in-hospital psychiatric disorder and maternal pre-gestation diabetes.
[a] Reference group was no PE no SGA no born <34 weeks of gestation after excluding maternal chronic hypertension and gestational hypertension.
[b] The analyses were adjusted for offspring birth year, sex, maternal age at delivery, mother's country of birth (Finland or not), mother married at birth (yes/no), maternal occupation (SES, upper white collar worker, lower white collar worker, blue collar worker, and other status), maternal smoking (yes/no), parity (0 or ≥1), maternal obesity (ICD-10: E65-E66) (yes/no), maternal gestational diabetes (yes/no), maternal out-patient psychiatric disorders (yes/no), maternal N05/N06 purchase during pregnancy, and maternal systemic inflammatory disease (yes/no).

© 2022 Kong L et al. *JAMA Network Open*.

eTable 12. Hazard Ratios for Offspring Neurodevelopmental and Psychiatric Disorders in Relation to Maternal Preeclampsia and Perinatal Complications

Maternal preeclampsia (PE) (*ICD-10* codes O11 or O14) and perinatal complications (SGA or delivery before 34 gestational weeks) (1 045 190 singleton live births 1996–2014 in Finland followed until 2018) not excluding births with in-hospital psychiatric disorder and maternal pre-gestational diabetes with insulin treatment.

PE stratified by SGA and gestational age[a]	Any F-diagnosis (n=100 000)	Psychotic disorders F20-F29 (n=3 203)	Mood disorders F30-39, F92 (n=41 656)	Anxiety disorders F40-43, F93 (n=54 969)	Eating disorders F50 (n=6 360)	Sleeping disorders F51 (n=6 586)	Personality disorders F60-69 (n=3 453)	Intellectual disabilities F70-79 (n=8 530)	Specific development al disorders F80-83 (n=58 859)	Autism spectrum disorders F84 (n=11 373)	ADHD& conduct disorders F90–91 (n=32 825)	Other behavioral& emotional disorders F98 (n=28 917)
**Crude model (Crude HR, 95% CI)**												
No PE no perinatal complications	1.00	1.00	1.00	1.00	1.00	1.00	1.00	1.00	1.00	1.00	1.00	1.00
PE without perinatal complications	1.16 (1.11-1.21)	0.85 (0.21-3.41)	1.15 (1.07-1.23)	1.20 (1.13-1.27)	1.17 (0.98-1.39)	1.23 (1.05-1.44)	0.99 (0.77-1.28)	1.28 (1.12-1.47)	1.30 (1.24-1.37)	1.45 (1.30-1.62)	1.90 (1.81-1.98)	1.94 (1.85-2.03)
PE with perinatal complications	2.06 (1.93-2.21)	1.63 (1.39-1.90)	1.21 (1.06-1.39)	1.36 (1.21-1.52)	1.44 (1.04-1.99)	0.85 (0.57-1.25)	1.93 (1.31-2.83)	3.37 (2.81-4.04)	2.78 (2.58-2.99)	1.96 (1.60-2.39)	1.35 (1.26-1.44)	1.23 (1.14-1.33)
Perinatal complications only	1.92 (1.87-1.97)	0.41 (0.15-1.09)	1.45 (1.38-1.52)	1.45 (1.39-1.51)	1.51 (1.34-1.70)	1.36 (1.21-1.53)	1.96 (1.69-2.26)	4.35 (4.08-4.65)	2.39 (2.32-2.47)	1.88 (1.74-2.04)	1.95 (1.73-2.20)	2.11 (1.87-2.38)
**Adjusted model[b] (Adjusted HR, 95% CI)**												
No PE no perinatal complications	1.00	1.00	1.00	1.00	1.00	1.00	1.00	1.00	1.00	1.00	1.00	1.00
PE without perinatal complications	1.17 (1.12-1.22)	2.32 (0.58-9.33)	1.11 (1.03-1.19)	1.12 (1.05-1.18)	1.12 (0.94-1.33)	1.15 (0.99-1.35)	0.99 (0.77-1.29)	1.26 (1.10-1.45)	1.23 (1.17-1.30)	1.23 (1.10-1.38)	1.22 (1.14-1.30)	1.12 (1.04-1.21)
PE with perinatal complications	2.08 (1.94-2.22)	1.42 (1.21-1.66)	1.21 (1.05-1.39)	1.29 (1.15-1.44)	1.31 (0.94-1.82)	0.79 (0.53-1.17)	1.86 (1.26-2.74)	3.40 (2.84-4.08)	2.78 (2.58-2.99)	1.66 (1.36-2.03)	1.90 (1.68-2.14)	2.01 (1.78-2.27)
Perinatal complications only	1.75 (1.71-1.80)	1.01 (0.38-2.69)	1.27 (1.21-1.33)	1.28 (1.22-1.33)	1.47 (1.31-1.66)	1.22 (1.09-1.38)	1.68 (1.46-1.94)	4.20 (3.93-4.48)	2.24 (2.17-2.31)	1.65 (1.52-1.78)	1.60 (1.53-1.68)	1.80 (1.72-1.90)

Preeclampsia was ICD-10 codes: O 11 or O14. Perinatal complications referred to SGA or delivery before 34 gestational weeks.
[a] Reference group was no PE, no SGA, and born ≥34 weeks of gestation after excluding maternal chronic hypertension and gestational hypertension.
[b] The analyses were adjusted for offspring birth year, sex, maternal age at delivery, mother's country of birth (Finland or not), mother married at birth (yes/no), maternal occupation (SES, upper white collar worker, lower white collar worker, blue collar worker, and other status), maternal smoking (yes/no), parity (0 or ≥1), maternal obesity (ICD-10: E65-E66, yes/no), maternal gestational diabetes (yes/no), maternal outpatient psychiatric disorders (yes/no), maternal N05/N06 purchase during pregnancy, and maternal systemic inflammatory disease (yes/no), maternal in-hospital psychiatric disorder and maternal pre-gestational diabetes with insulin treatment.

© 2022 Kong L et al. *JAMA Network Open*.

eTable 13. Hazard Ratios for Offspring Neurodevelopmental and Psychiatric Disorders in Relation to Gestational Hypertension and Perinatal Complications

Gestational hypertension (*ICD-10* code O13) and perinatal complications (SGA or delivery before 34 gestational weeks)

GH stratified by SGA and gestational age[a]	Any F-diagnosis (n=99 368)	Psychotic disorders F20-F29 (n=3 195)	Mood disorders F30-39, F92 (n=41 410)	Anxiety disorders F40-43, F93 (n=54 636)	Eating disorders F50 (n=6 301)	Sleeping disorders F51 (n=6 569)	Personality disorders F60-69 (n=3 420)	Intellectual disabilities F70-79 (n=8 506)	Specific development al disorders F80-83 (n=58 602)	Autism spectrum disorders F84 (n=11 320)	ADHD& conduct disorders F90–91 (n=32 614)	Other behavioral& emotional disorders F98 (n=28 730)
**Crude model (Crude HR, 95% CI)**												
No GH, no perinatal complications	1.00	1.00	1.00	1.00	1.00	1.00	1.00	1.00	1.00	1.00	1.00	1.00
GH, no perinatal complications	0.80 (0.74-0.86)	0.54 (0.27-1.09)	0.79 (0.68-0.92)	1.01 (0.90-1.12)	0.64 (0.39-1.05)	0.94 (0.73-1.21)	0.39 (0.17-0.87)	1.16 (0.95-1.40)	1.23 (1.15-1.32)	1.19 (1.01-1.41)	1.23 (1.11-1.37)	1.08 (0.97-1.21)
GH with perinatal complications	1.50 (1.25-1.79)	1.64 (0.41-6.58)	1.20 (0.77-1.86)	1.08 (0.75-1.56)	NA	3.13 (1.94-5.04)	1.56 (0.39-6.23)	2.77 (1.81-4.25)	2.15 (1.78-2.59)	1.37 (0.79-2.35)	2.13 (1.62-2.79)	1.71 (1.26-2.31)
Perinatal complications only	1.91 (1.87-1.97)	1.56 (1.33-1.84)	1.44 (1.38-1.51)	1.45 (1.39-1.51)	1.50 (1.33-1.68)	1.33 (1.19-1.50)	1.97 (1.71-2.27)	4.33 (4.07-4.62)	2.39 (2.31-2.46)	1.88 (1.74-2.03)	1.90 (1.81-1.98)	1.94 (1.85-2.04)
**Adjusted model [b] (Adjusted HR, 95% CI)**												
No GH, no perinatal complications	1.00	1.00	1.00	1.00	1.00	1.00	1.00	1.00	1.00	1.00	1.00	1.00
GH, no perinatal complications	1.08 (1.01-1.16)	1.36 (0.68-2.74)	0.90 (0.77-1.05)	0.98 (0.87-1.09)	0.78 (0.48-1.28)	0.95 (0.74-1.23)	0.75 (0.34-1.68)	1.28 (1.06-1.56)	1.14 (1.06-1.22)	1.08 (0.91-1.27)	1.07 (0.97-1.19)	1.00 (0.90-1.12)
GH with perinatal complications	2.00 (1.67-2.40)	3.98 (0.99-15.97)	1.37 (0.88-2.13)	1.04 (0.72-1.50)	NA	3.09 (1.92-4.98)	3.00 (0.75-12.04)	3.12 (2.03-4.79)	1.99 (1.65-2.40)	1.14 (0.66-1.96)	1.77 (1.35-2.32)	1.58 (1.17-2.14)
Perinatal complications only	1.75 (1.71-1.80)	1.36 (1.16-1.60)	1.27 (1.21-1.33)	1.28 (1.23-1.33)	1.47 (1.30-1.65)	1.20 (1.07-1.36)	1.70 (1.48-1.96)	4.17 (3.91-4.45)	2.24 (2.17-2.31)	1.63 (1.51-1.77)	1.61 (1.53-1.68)	1.82 (1.73-1.91)

GH, gestational hypertension referred to ICD-10 code: O13. Perinatal complications referred to SGA or delivery before 34 gestational weeks. NA, not applicable.
Exclusion: Maternal preeclampsia (ICD-10 codes: O11 or O14).
1 045 447 singleton live births 1996–2014 in Finland followed until 2018) not excluding births with in-hospital psychiatric disorder and maternal pre-gestational diabetes with insulin treatment
[a] Reference group was no GH, no SGA, and born ≥34 weeks of gestation.
[b] The analyses were adjusted for offspring birth year, sex, maternal age at delivery, mother's country of birth (Finland or not), mother married at birth (yes/no), maternal occupation (SES, upper white collar worker, lower white collar worker, blue collar worker, and other status), maternal smoking (yes/no), parity (0 or ≥1), maternal obesity (ICD-10: E65-E66, yes/no), maternal gestational diabetes (yes/no), maternal outpatient psychiatric disorders (yes/no), maternal N05/N06 purchase during pregnancy, maternal systemic inflammatory disease (yes/no), maternal in-hospital psychiatric disorder (yes/no), and maternal pre-gestational diabetes (yes/no).

© 2022 Kong L et al. *JAMA Network Open*.

eTable 14. Mediating Effect of Perinatal Complications in the Association Between Maternal Preeclampsia and Any Offspring *ICD-10* F Diagnosis

Hazard ratio (HR) and 95 % CI from mediation models.

**Maternal preeclampsia**	**HR[a]**	**95% CI**
Total association	1.05	1.04-1.07
Direct association	1.01	1.00-1.03
Mediating effect by perinatal complications	1.04	1.02-1.07

Preeclampsia referred to ICD-10 code: O11 or O14. Perinatal complications referred to SGA or delivery before 34 gestational weeks.
Exclusion: Maternal in-hospital psychiatric disorder and maternal pre-gestational diabetes.
[a]The analyses were adjusted for offspring birth year, sex, maternal age at delivery, mother's country of birth (Finland or not), mother married at birth (yes/no), maternal occupation (SES, upper white collar worker, lower white collar worker, blue collar worker, and other status), maternal smoking (yes/no), parity (0 or ≥1), maternal obesity (ICD-10: E65-E66, yes/no), maternal gestational diabetes (yes/no), maternal outpatient psychiatric disorders (yes/no), maternal N05/N06 purchase during pregnancy, and maternal systemic inflammatory disease (yes/no).

© 2022 Kong L et al. *JAMA Network Open*.

eFigure 1. Risks of Offspring Psychotropic Medication Purchase In Relation to Categories of Maternal Preeclampsia and Perinatal Complications

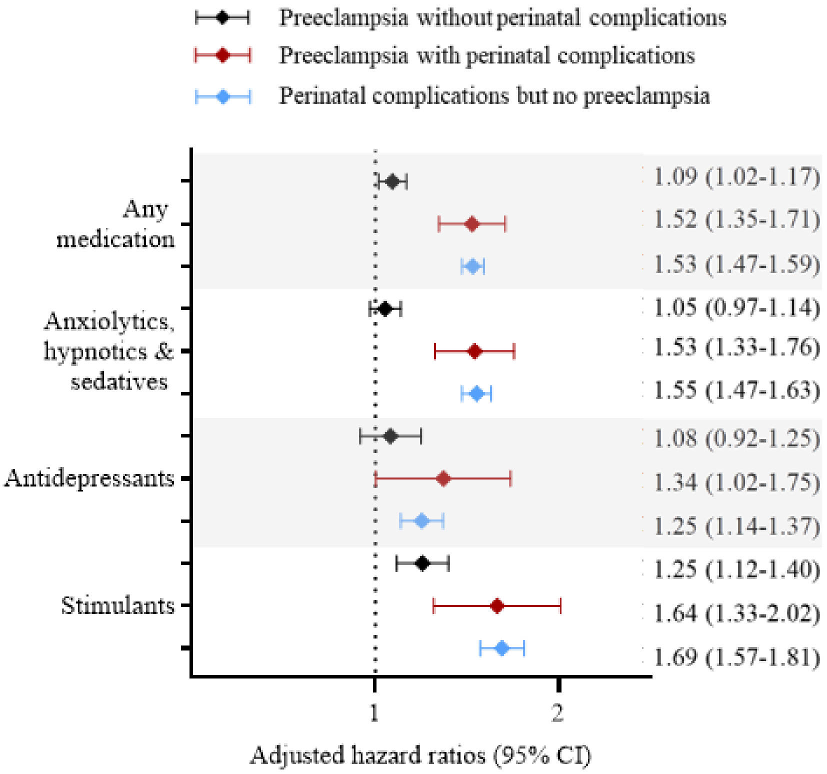

Reference group was no preeclampsia, no SGA, and born after 33 gestational weeks after excluding maternal chronic hypertension and gestational hypertension. The analyses were adjusted for offspring birth year, sex, and the maternal factors age at delivery, country of birth (Finland or not), married at birth (yes/no), occupation (upper white collar worker, lower white collar worker, blue collar worker, and other status), smoking (yes/no), parity (0 or ≥1), obesity (ICD-10: E65-E66, yes/no), gestational diabetes (yes/no), outpatient psychiatric disorders (yes/no), N05/N06 purchase during pregnancy (yes/no), and systemic inflammatory disease (yes/no). All children were followed up until December 2018.

© 2022 Kong L et al. *JAMA Network Open*.

eFigure 2. Mediating Effect of Perinatal Complications in the Association Between Maternal Preeclampsia and Any Offspring *ICD-10* F Diagnosis

Regression coefficient (SE) derived from the mediation model. Corresponds to eTable 14.

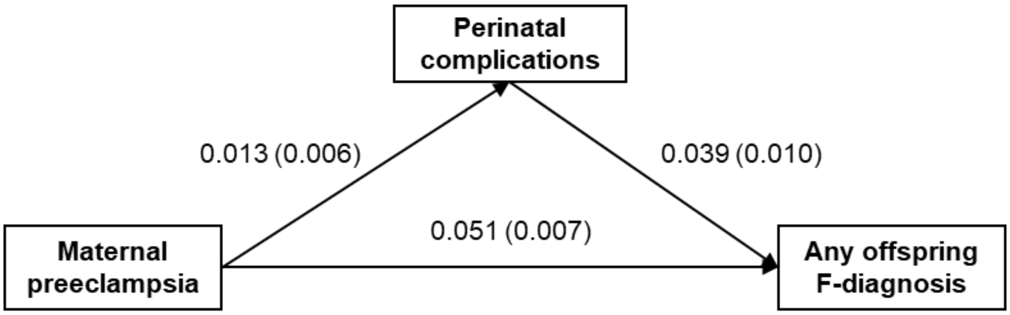

Preeclampsia referred to ICD-10 code: O11 or O14. Perinatal complications referred to SGA or delivery before 34 gestational weeks. Exclusion: Maternal in-hospital psychiatric disorder and maternal pre-gestational diabetes.
This model was adjusted for offspring birth year, sex, maternal age at delivery, mother's country of birth (Finland or not), mother married at birth (yes/no), maternal occupation (SES, upper white collar worker, lower white collar worker, blue collar worker, and other status), maternal smoking (yes/no), parity (0 or ≥1), maternal obesity (ICD-10: E65-E66, yes/no), maternal gestational diabetes (yes/no), maternal outpatient psychiatric disorders (yes/no), maternal N05/N06 purchase during pregnancy, and maternal systemic inflammatory disease (yes/no).

© 2022 Kong L et al. *JAMA Network Open*.
